# Synergistic augmentation of rhythmic myogenic contractions of human stomach by arginine vasopressin and adrenaline: Implications for the induction of nausea

**DOI:** 10.1111/bph.15943

**Published:** 2022-09-22

**Authors:** Raj Makwana, Ellie Crawley, Marilisa Straface, Alexandra Palmer, Armen Gharibans, Kalpana Devalia, John Loy, Greg O'Grady, Paul L. R. Andrews, Gareth J. Sanger

**Affiliations:** ^1^ Blizard Institute, Faculty of Medicine and Dentistry Queen Mary University of London London UK; ^2^ Department of Surgery and Auckland Bioengineering Institute University of Auckland Auckland New Zealand; ^3^ Bariatric Surgery Department Homerton University Hospital London UK; ^4^ Division of Biomedical Sciences St George's University of London London UK

**Keywords:** arginine vasopressin, adrenaline, human, interstitial cells of Cajal, nausea, stomach

## Abstract

**Background and Purpose:**

Nausea is associated with the hormonal secretion of vasopressin and adrenaline, although their actions in inducing nausea is poorly understood. Here, we have investigated their actions on human stomach muscle.

**Experimental Approach:**

Muscle strips were suspended in tissue baths and neuronal‐/non‐neuronally‐mediated contractions were measured. Custom software analysed eight motility parameters defining spontaneous phasic non‐neuronally mediated contractions. Receptor distributions were assessed by qPCR and immunofluorescence.

**Key Results:**

V_1A_ receptors and α_1_‐adrenoceptors were located on muscle as well as interstitial cells of Cajal (ICCs). Myogenic contractions of human proximal and distal stomach (respectively, 2.6 ± 0.1 and 2.7 ± 0.0 per minute; n = 44) were larger in the distal area (1.1 ± 0.1 and 5.0 ± 0.1 mN), developing relatively slowly (proximal) or rapidly (distal). Vasopressin caused tonic (proximal) or short‐lived (distal) increases in muscle tone and increased myogenic contraction amplitude, frequency and rate (acting at V_1A_ receptors; thresholds 10^−11^–10^−10^ M); by contrast, cholinergically mediated contractions were unaffected. Oxytocin acted similarly to vasopressin but less potently, at OT receptors). Adrenaline increased (10^−10^–10^−5^ M; α_1_‐adrenoceptors) and decreased (≥10^−6^ M; β‐adrenoceptors) muscle tone and enhanced/reduced myogenic contractions. Cholinergically mediated contractions were reduced (α_2_‐adrenoceptors). Combined, vasopressin (10^−9^ M) and adrenaline (10^−8^ M) increased muscle tone and phasic myogenic activity in a synergistic manner.

**Conclusions and Implications:**

Vasopressin and adrenaline increased human gastric tone and myogenic contraction amplitude, rate of contraction and frequency. In combination, their actions were further increased in a synergistic manner. Such activity may promote nausea.

**Abbreviations**
AP: area postrema
AVP: arginine vasopressin
EGG: electrogastrography
GAPDH: glyceraldehyde 3‐phosphate dehydrogenase
ICCs: interstitial cells of Cajal
l‐NAME: Nω‐nitro‐l‐arginine methyl ester hydrochloride
OT: oxytocin

**What is already known**

- Area postrema activation by arginine vasopressin is considered the mechanism by which it induces vomiting.
- Elevated levels of plasma AVP and adrenaline accompany nausea and gastric electrical dysrhythmia.

**What does this study add**

- Spontaneous, non‐neuronally mediated muscle contractions were larger and developed faster in the human stomach antrum.
- Low concentrations of vasopressin and adrenaline acted synergistically to increase muscle tone and myogenic activity.

**What is the clinical significance**

- Synergy of motor activity between low concentrations of vasopressin and adrenaline in stomach increases impact.
- Signalled to the brain, such gastric motor activity may promote nausea in humans.

## 1 INTRODUCTION

The unpleasant self‐reported experience of nausea (Stern et al., 2011) often precedes vomiting and seems less effectively treated than vomiting (Sanger & Andrews, 2018). This experience includes patients receiving anti‐cancer chemotherapy (Smit et al., 2021). Drug treatments also fail to adequately alleviate nausea in gastroparesis, chronic unexplained nausea and vomiting and functional dyspepsia (Chedid et al., 2018; Parkman et al., 2016). Thus, the pathways of nausea and vomiting can overlap, but differences do exist, thus complicating treatments (Sanger & Andrews, 2018; Stern et al., 2011).

Diverse physiological changes can accompany nausea, including heightened sympathetic and reduced parasympathetic activity (LaCount et al., 2011), pituitary secretion of arginine vasopressin (AVP) (Koch et al., 1990), and adrenaline and cortisol from adrenal glands (Xu et al., 1993). Additionally, dysrhythmic gastric electrical activity (Koch, 1997) and antral hypomotility (Faas et al., 2001) can occur. A key challenge is to determine which of these changes contributes to the development of nausea and which represents a physiological response to the stress of nausea.

AVP is of particular interest (Stern et al., 2011). In humans, AVP enhances renal water reabsorption, increases peripheral vascular resistance (Holt & Haspel, 2010) and is associated with nausea in several clinical paradigms (e.g., motion sickness, water intoxication and tilt‐induced syncope; Heyer et al., 2017; Rowe et al., 1979) in addition to vomiting in humans (Edwards et al., 1989) and animals (Verbalis et al., 1987). Further, intravenously administered AVP (and its analogues) can induce nausea without vomiting or retching (Caras et al., 1997; Kim et al., 1997). It is assumed that systemic AVP induces vomiting by activating neurons in the area postrema (AP), also known as the ‘chemoreceptor trigger zone’. However, the evidence relies mostly on iontophoretic application of AVP activating 50% of AP neurons tested in dogs (Carpenter et al., 1988), at a concentration (10^−3^ M) which undiluted can activate all AVP receptors, as well as oxytocin (OT) receptors (Bichet et al., 2019). This broad activity has not been causally linked to vomiting, or to the other cardiovascular, feeding and metabolic functions of the AP (Price et al., 2008).

Some evidence links nausea induced by systemic AVP with changed dominant frequency of gastric electrical slow waves (assessed by electrogastrography [EGG]), either by creating tachygastria (Caras et al., 1997; Kim et al., 1997) or bradygastria (Kim et al., 1997; Lien et al., 2003). This evidence finds some consistency with EGG studies that have demonstrated gastric dysrhythmia (tachygastria and/or bradygastria) before and/or during nausea induced by different stimuli (Heyer et al., 2017; Koch, 1997; Koch et al., 1990). The gastric motor correlates are not fully described, but tachygastria has been linked with antral hypomotility (Ouyang et al., 2005). Reduced gastric contraction amplitude (particularly in the antrum; Chen et al., 1993) and delayed emptying are associated with nausea (Faas et al., 2001).

It is 25 years since the ‘noxious trio’ of nausea, gastric dysrhythmia and AVP was described (Koch, 1997), yet remarkably, AVP has only recently been shown to directly stimulate human stomach contractility (contrasting with inactivity of copeptin, a glycosylated peptide from the C‐terminus of the AVP precursor, co‐secreted with AVP; Makwana et al., 2021). Accordingly, the present study has several inter‐linked objectives: (i) characterize the spontaneous, non‐neuronally mediated ‘myogenic’ contractions of human stomach (initiated by depolarization of interstitial cells of Cajal [ICCs]; Sanders et al., 2014) and compare with published measurements of gastric electrical activity recorded in vivo; (ii) fully characterize the effects of AVP on human stomach muscle tone, myogenic and neuronally mediated contractions, defining the receptor involved and clinically contextualizing by comparing active concentrations with those reported in plasma during nausea; (iii) investigate the activity of adrenaline (ADr) released with AVP during nausea (Hasler et al., 1995; Heyer et al., 2017; LaCount et al., 2011) and known to be involved in the accompanying disrupted gastric slow wave cycling (Chen et al., 1993); could AVP and ADr act together to disrupt gastric movements, reaching a ‘tipping point’ at which nausea is perceived?; and (iv) determine if receptors for AVP and ADr are localized to ICCs. These objectives address the fundamental question: Could low concentrations of AVP and ADr signal nausea by directly influencing gastric motility?

## 2 METHODS

### 2.1 Human stomach

Resected sections of human stomach were obtained from obese donors (12:41 male:female; 23–67 [median 48] years) undergoing sleeve gastrectomy (cut ~2 cm distal to gastro‐oesophageal junction, ~8 cm proximal to pylorus, ~⅔rd of width from greater curvature). Table S1 summarizes patient demographics, including any use of current medications (these were varied and did not include GLP‐1 analogues). Tissues were used as they became available, without regard to donor sex, age or body mass index, but some retrospective analysis of patient characteristics was performed. Ethical approval (REC‐15/LO/2127) and written informed consent were obtained. Sections ~5 cm^2^ were cut ~5 cm from both ends of the stomach, excluding areas that were damaged during surgery and during transport in the Krebs–Henseleit solution (×10^−3^ M: NaCl 118.3, KCl 4.7, MgSO_4_ 1.2, KH_2_PO_4_ 1.2, NaHCO_3_ 25, d‐glucose 11.1, CaCl_2_ 2.5) pre‐gassed with 95% O_2_/5% CO_2_. The resected areas most likely represented proximal corpus and distal corpus–proximal antrum, respectively, and, for brevity, are defined as ‘proximal’ and ‘distal’ stomach. Methods for minimizing variation were followed when using human gastrointestinal (GI) tissue (Sanger et al., 2013).

### 2.2 Functional experiments

Methods have been described elsewhere (Makwana et al., 2021; Straface et al., 2022). Briefly, mucosa‐free muscle strips (5 × 20 mm) were cut (perpendicular to greater curvature, parallel to circular muscle fibres) and either used immediately or after overnight storage (4°C in the Krebs–Henseleit solution). In preliminary experiments, storage did not appear to affect contractile activity (Table S2), confirming previous studies (Broad et al., 2012), and data were pooled.

Strips were mounted in tissue baths to isometric force transducers (MLT201/D, ADInstruments, UK) between platinum wire electrodes and bathed in the Krebs–Henseleit solution (95% O_2_/5% CO_2_) maintained at 37°C; changes in muscle tone were recorded (milliNewtons [mN]) using the AcqKnowledge v3.8.1 data acquisition system (BIOPAC Systems, USA) on personal computers (www.dell.com/uk). After stretching (20 mN) and equilibration, strips were treated with a test substance for 30 min before constructing cumulative concentration–response curves for AVP, oxytocin (OT) or ADr. Non‐cumulative AVP concentration–response curves were constructed separately.

During electrical field stimulation (EFS; 5 Hz frequency, 0.5 ms pulse width; for 10 s·min^−1^; voltage 10% greater than required to elicit maximal contractions; electrodes connected to STG2008 stimulators: Multi Channel Systems, Germany), test substances were applied for 30 min before constructing cumulative concentration–response curves for AVP, OT or ADr (log_10_‐unit increments with 15 min intervals or when effect of previous concentration peaked), with one curve/tissue. Treatments were randomized between tissue baths and paired with time‐matched vehicle‐treated controls.

After completing an experiment, muscle strips were challenged with carbachol, 10^−3^ M, to estimate maximal contractile capacity.

#### 2.2.1 Expression of data

**FIGURE 1:**
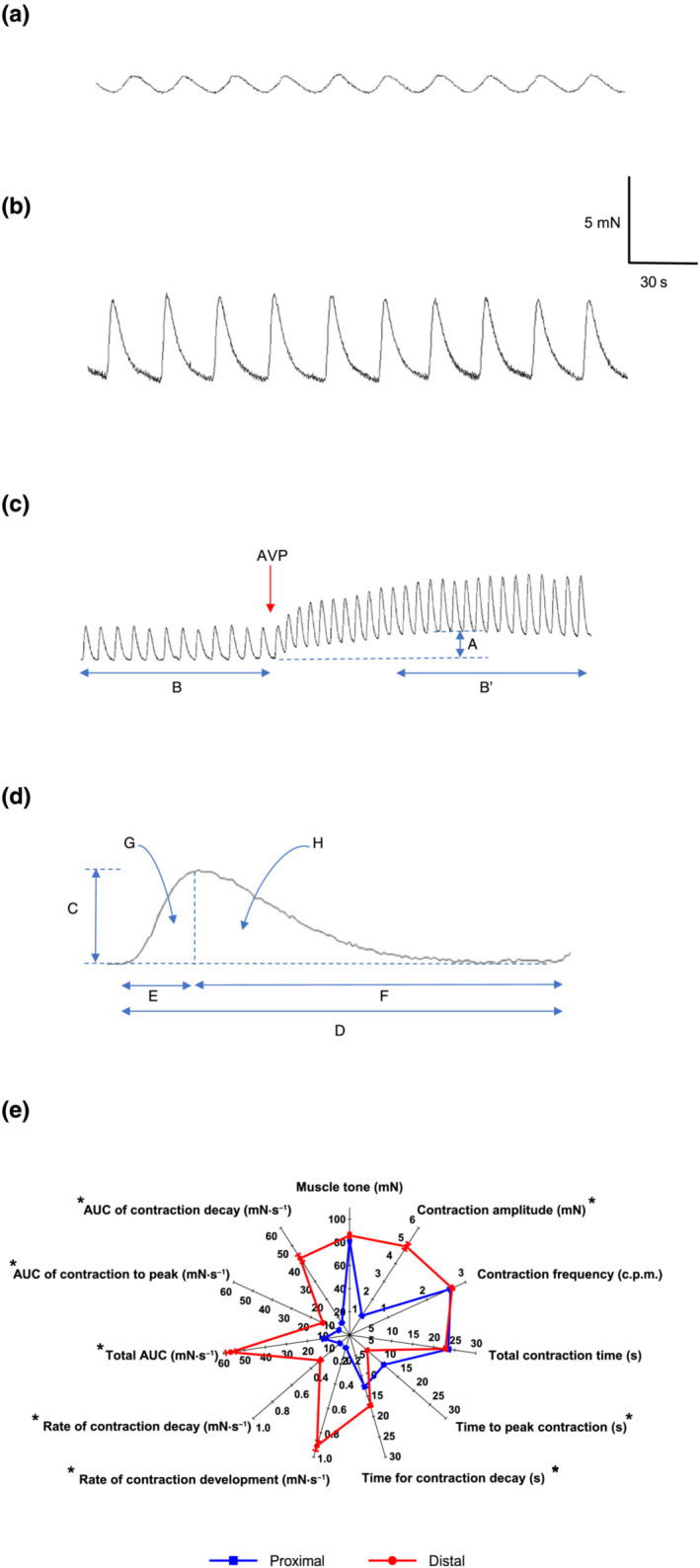
Spontaneous contractions of human (a) proximal and (b) distal stomach circular muscle. (c) Spontaneous contractions of distal stomach and change in A = muscle tone (mN) and frequency of contractions (number of contractions per minute [c.p.m.]), usually measured over 5 min B = before and B′ = after application of an agonist (e.g., arginine vasopressin [AVP]). (d) Features measured of a given contraction wave, C = contraction amplitude (mN), D = total contraction time (s), E and F = time for contraction to peak and decay (s), G and H = area under the curve for contraction to peak and decay (mN·s^−1^). G and H = total area under curve (mN·s^−1^), mean change in C over E, and C over F = rates of contraction development and decay (mN·s^−1^). (e) Radar plot comparing features of the baseline contraction waveform of proximal and distal stomach. Also shown is the carbachol (10^−3^ M)‐induced maximal increase in muscle tone. *P < 0.05 between data sets. Data are mean ± SEM. n = 44

Spontaneous contractions were quantified using custom software (https://github.com/agharibans/GISMCA), measuring change in baseline muscle tone (mN), amplitude of contractions (mN), number of contractions per minute (c.p.m.) during steady state, total contraction time (s), time for contractions to peak and decay (s), rates of contraction development and decay (mN·s^−1^) and areas under the contraction wave (AUC) and during the development and decay phases (mN·s^−1^) (Figure 1c–e). Measurements were visualized as radar plots using OriginPro 2020 (OriginLab Corporation, USA).

Agonist‐induced increases in baseline muscle tone were expressed as percentage of carbachol‐evoked contraction. For experiments with EFS, the effects of agonists were quantified as percentage change in amplitude of EFS‐evoked contractions immediately before first addition.

Individual agonist concentration–response curves were fitted by non‐linear regression. Ratios for increased agonist *p*EC_50_ in the presence of increasing concentrations of antagonist were analysed using the Schild plots, determining the *p*K_B_ of the antagonist–receptor complex. When using single concentrations of antagonist, the potency (*p*A_2_) was determined by the Gaddum–Schild equation (see Methods S1 for more details about the curve fitting and data manipulation).

#### 2.2.2 Materials

Ascorbic acid, atropine sulfate, carbachol, cromolyn sodium, l‐NAME (Nω‐nitro‐l‐arginine methyl ester hydrochloride), TTX (tetrodotoxin) and salts for the Krebs–Henseleit solution were supplied by Sigma‐Aldrich (Gillingham, UK). AVP, OT, SR49059 (V_1A_ receptor antagonist; Serradeil‐Le Gal et al., 1993) and L371257 (OT receptor antagonist; Griffante et al., 2005) were supplied by Tocris Biosciences (Abingdon, UK). ADr (l‐epinephrine) and yohimbine hydrochloride were supplied by Fisher Scientific (Loughborough, UK). Propranolol hydrochloride was supplied by Insight Biotechnology (London, UK). Mepyramine maleate and prazosin hydrochloride were supplied by Cambridge Bioscience (Cambridge, UK). Ascorbic acid, AVP, carbachol, l‐NAME, OT and TTX were dissolved in water; stock/dilutions of ADr in ascorbic acid (10^−1^ M); and other drugs in 100% dimethyl sulfoxide (DMSO). Total volume of solvents added to tissue baths was not >1% bath volume.

### 2.3 Receptor expression and localization

Stomach muscle ~0.5 cm^2^ (n = 8; 4:4 male:female; 2/8 diabetic; 28–54 [median 41] years) was stored at −80°C in RNAlater™, and receptor mRNA expression was determined by qPCR (Methods S1).

Receptor localization was investigated by immunofluorescence (3:3 male:female; 3/6 diabetic, 40–59 [median 47] years; Methods S1). Full‐thickness distal stomach (~1 cm^2^) was fixed in 10% formalin and embedded in paraffin wax. Transverse sections (4 μm thickness) were deparaffinized, rehydrated and subjected to heat‐mediated antigen retrieval before incubation with optimized concentrations of primary antibody overnight at 4°C. Subsequently, sections were incubated with secondary antibodies for 1 h after epitope blockade with 1% bovine serum albumin (BSA), followed by an auto‐fluorescence quencher (Vector® TrueVIEW® Autofluorescence Quenching Kit; Vector Laboratories) and nuclei counterstain 4′,6‐diamidino‐2‐phenylindole (DAPI) at room temperature. Coverslips were mounted (VECTASHIELD Vibrance Antifade Mounting; Vector Laboratories, USA) before imaging (Olympus BX61 microscope; Olympus, UK), SmartCapture3 (DSUK, UK) and analyses (ImageJ; https://imagej.nih.gov/ij/). The immuno‐related procedures used comply with the recommendations made by the British Journal of Pharmacology (Alexander et al., 2018).

### 2.4 Statistical analysis

The data and statistical analysis comply with the recommendations of the *British Journal of Pharmacology* on experimental design and analysis in pharmacology (Curtis et al., 2018). Data were quantified as mean ± standard error of the mean (SEM). In functional studies, paired or unpaired Student's *t* tests compared individual means of data from a given stomach or between stomachs from different donors, respectively. Shifts of agonist concentration–response curves by test ligands were compared by one‐way analysis of variance (ANOVA) followed by Dunnett's post hoc test for multiple comparisons. *P* < 0.05 represented statistical significance. *n* values represent number of patients. Only one muscle strip was used per drug treatment from a given patient.

Inter‐rater agreement between two independent observers assessing immunofluorescence of a given receptor was investigated using Bland–Altman plots.

### 2.5 Nomenclature of targets and ligands

Key protein targets and ligands are hyperlinked to corresponding entries in http://www.guidetopharmacology.org, the common portal for data from the IUPHAR/BPS Guide to PHARMACOLOGY, permanently archived in the Concise Guide to PHARMACOLOGY 2020/21. Key family targets are G protein‐coupled receptors (Alexander et al., 2021).

## 3 RESULTS

### 3.1 Receptor expression

Relative to glyceraldehyde 3‐phosphate dehydrogenase (GAPDH) expression (similar in both stomach regions), V_1A_ , V_1B_ , V_2_ , OT and adrenergic α_1A_ , α_1B_ , α_1D_ , α_2A–C_, β_1–3_ receptor mRNA were present in muscle of both regions (Figure S1). There was no clear difference between results from donors with or without diabetes and these were combined. Rank orders of expression for the two families were V_1A_ > V_1B_ = V_2_ = OT and β_2_ = β_3_ > α_1D_ = α_1B_ > α_2A_ = α_2C_ > β_1_ = α_2B_ = α_1A_.

### 3.2 Spontaneous contractions

Proximal and distal stomach muscle displayed spontaneous phasic contractions (respectively, 1.1 ± 0.1 and 5.0 ± 0.2 mN; n = 44); the former was 5× smaller than the latter (Figure 1a,b,e). These and other measurements were independent of patients' sex or diabetic status (Figure S2); no patient was diagnosed with diabetic gastroparesis, a condition associated with damaged ICCs (Ördög, 2008) (Table S1). The duration of individual contractions was similar (respectively, 23.5 ± 0.4 and 22.8 ± 0.3 s), providing matching rhythmic frequencies (2.6 ± 0.1 and 2.7 ± 0.1 c.p.m.). Correspondingly, contractions of proximal stomach developed and decayed with comparable rates whereas those of distal stomach peaked ~5× faster and decayed more slowly.

**FIGURE 2:**
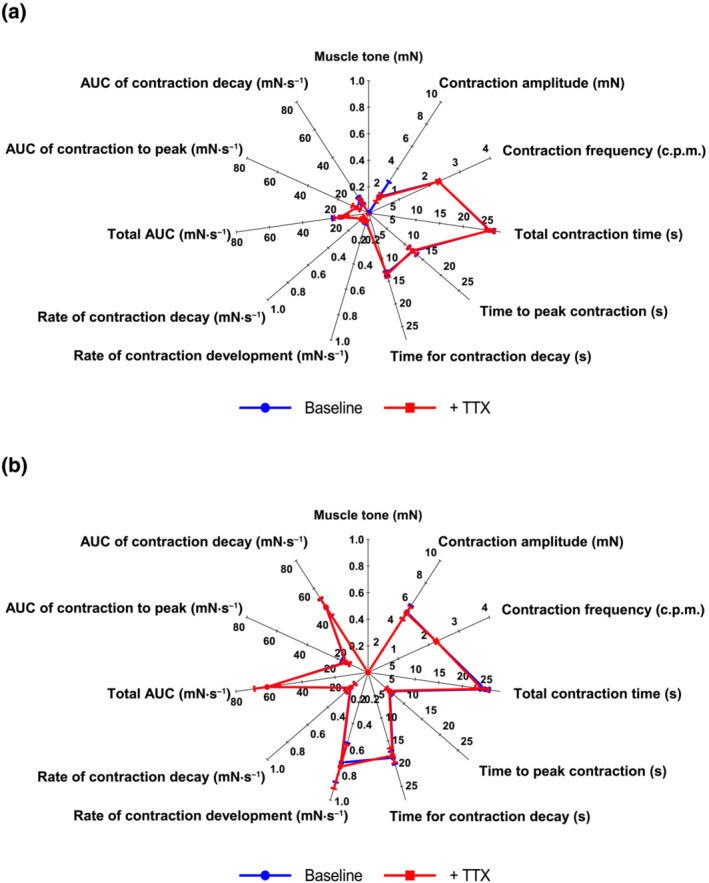
Comparison between the various features of the spontaneous contraction waveform of human (a) proximal and (b) distal stomach circular muscle before (baseline) and after 30 min incubation with tetrodotoxin (TTX; 10^−6^ M). Each value represents the mean ± SEM. n = 4

TTX (10^−6^ M) did not alter baseline muscle tone or spontaneous contractions (Figure 2). Atropine (10^−6^ M) alone or with TTX and l‐NAME (3 × 10^−4^ M) caused small tone reductions in proximal and distal stomach (respectively, by 1.6 ± 0.2 and 1.4 ± 0.1 mN, n = 6 both) compared with time‐matched controls but had no effect on spontaneous contractions (Figure S3).

Carbachol produced similar maximal increases in muscle tone in both stomach regions (Figure 1e).

### 3.3 AVP and OT

#### 3.3.1 Muscle tone and spontaneous contractions

Concentration–response curves for AVP were similar when constructed using single concentrations/muscle strip or by cumulative application, suggesting minimal tachyphylaxis (Figure S4A–C and Table S3). Subsequent experiments used cumulative application. Notably, low single concentrations of AVP (≤10^−9^ M) evoked slow, sustained contractions and increases in spontaneous contractions, whereas higher concentrations caused shorter lived responses (Figure S4D).

**FIGURE 3:**
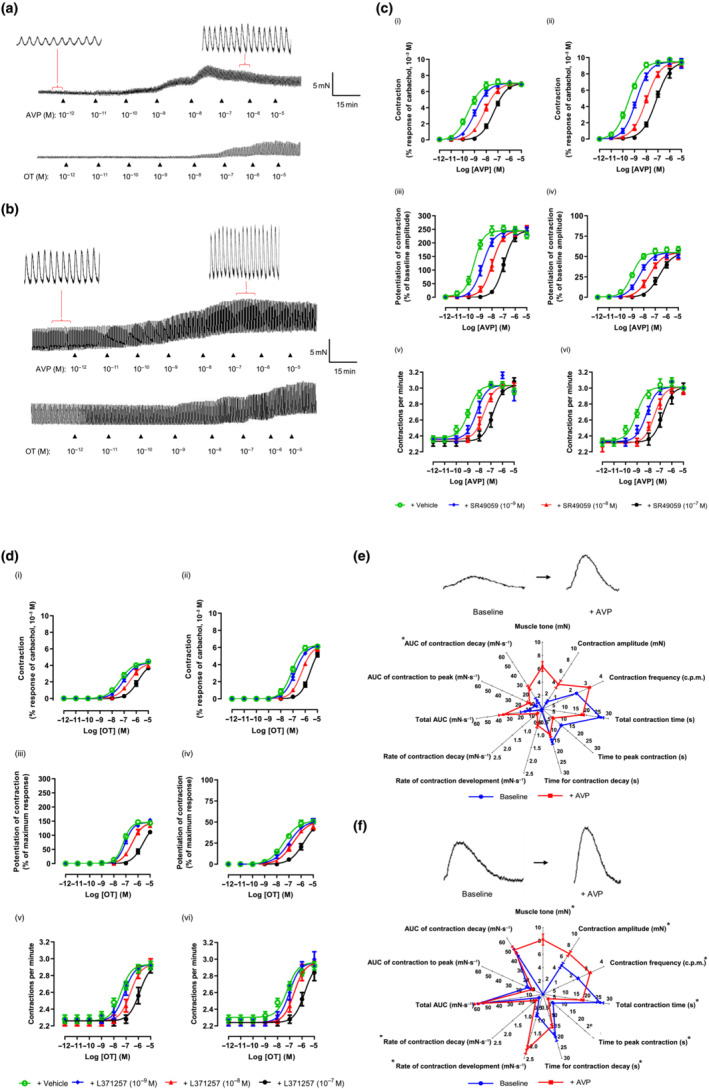
Effects of arginine vasopressin (AVP) and oxytocin (OT) on spontaneous contractions of human (a) proximal and (b) distal stomach circular muscle. (c) AVP‐ and (d) OT‐induced increase in muscle tone (i, ii) and amplitude (iii, iv) and frequency (v, vi) of spontaneous contractions of human proximal (graphs on left) and distal (right) stomach, after 30 min incubation with SR49059 and L371257, respectively, or vehicle (dimethyl sulfoxide [DMSO] 0.1% v/v). n = 5. (e) and (f) compare a spontaneous contraction of human (a) proximal and (b) distal stomach before (baseline) and after a single concentration of AVP (10^−7^ M), together with the AVP‐induced increase in muscle tone. n = 4. Data are mean ± SEM

AVP concomitantly increased muscle tone, amplitude and frequency of contractions in both stomach regions (Figure 3). OT acted similarly but was less potent and effective than AVP (Figure 3). The potency and threshold concentrations of both hormones were similar in each stomach region (Table 1a). The maximum increase in baseline muscle tone by AVP and OT was greater in distal, compared with proximal, stomach. Further, both caused larger increases in muscle tensions developed by the larger phasic contractions of distal stomach, relative to the smaller contractions of proximal stomach (although % increase greater in proximal stomach). AVP (10^−7^ M) increased the frequency of contractions by respectively increasing and decreasing the rate and duration of contraction development and decay. Coupled to the increased amplitude of contraction, this resulted in similar changes in ‘area under the curve’ for each phase of contraction (Figure 3e,f). TTX (10^−6^ M) had no effect on AVP‐ and OT‐induced responses (Table S4 and Figure S5). Atropine (10^−6^ M) and its combination with TTX and l‐NAME (3 × 10^−4^ M), previously shown to cause small reductions in baseline muscle tone, caused small decreases in potency of AVP and OT (Table S4 and Figure S5). Finally, in preliminary experiments, the response to AVP was unaffected by the mast cell stabilizer cromolyn (10^−5^ M) or the histamine H_1_ receptor antagonist mepyramine (10^−7^ M) (Figure S6 and Table S5), which alone did not appear to affect baseline muscle tone and spontaneous contractions.

**TABLE 1:** Threshold concentration, potency (*p*EC_50_) and maximal effect (E_max_) of (a) AVP and OT, for increasing muscle tone, amplitude and frequency of spontaneous contractions of human proximal and distal stomach circular muscle; (b) data for effect of adrenaline on spontaneous and EFS‐evoked contractions of human proximal and distal stomach

(a)												
	Proximal						Distal					
	AVP			OT			AVP			OT		
Effect	Threshold (M)	*p*EC_50_	E_max_ (%)	Threshold (M)	*p*EC_50_	E_max_ (%)	Threshold (M)	*p*EC_50_	E_max_ (%)	Threshold (M)	*p*EC_50_	E_max_ (%)
**Tone**	10^−11^–10^−10^	9.5 ± 0.2	7.1 ± 0.2	10^−9^–10^−8^	7.5 ± 0.0	4.4 ± 0.1	10^−11^–10^−10^	9.5 ± 0.1	9.5 ± 0.2	10^−9^–10^−8^	7.1 ± 0.1	6.3 ± 0.12
**Amplitude**	10^−11^–10^−10^	9.5 ± 0.1	244.8 ± 3.9	10^−8^	7.2 ± 0.1	145.6 ± 2.3	10^−10^	9.0 ± 0.1	55.0 ± 2.0	10^−9^–10^−8^	7.4 ± 0.1	51.5 ± 1.5
**Frequency**	10^−11^–10^−10^	8.9 ± 0.1	30.4 ± 2.6	10^−9^–10^−8^	7.4 ± 0.1	30.0 ± 2.6	10^−11^–10^−10^	9.0 ± 0.1	33.6 ± 1.0	10^−9^–10^−8^	7.2 ± 0.2	30.5 ± 2.5

(b)					
		Proximal		Distal	
Experimental condition	Effect	*p*EC_50_	E_max_ (%)	*p*EC_50_	E_max_ (%)
**Baseline conditions**	**Tone (contraction)**	9.5 ± 0.2	6.3 ± 0.5	9.3 ± 0.1	7.6 ± 0.5
	**Tone (relaxation)**	5.1 ± 0.2	100	5.1 ± 0.2	100
	**Amplitude**	8.6 ± 0.1	244.3 ± 10.2	8.7 ± 0.1	56.4 ± 3.8
	**Frequency**	7.6 ± 0.4	23.4 ± 1.9	8.1 ± 0.4	25.2 ± 1.2
**EFS conditions**	**Tone (contraction)**	8.9 ± 0.1	5.1 ± 0.3	9.0 ± 0.1	6.4 ± 0.4
	**Tone (relaxation)**	5.4 ± 0.2	100	4.8 ± 0.1	100
	**EFS‐evoked contraction inhibition**	5.9 ± 0.2	58.8 ± 4.9	6.2 ± 0.1	73.4 ± 4.0

*Note*: Data are mean ± SEM. (a) n = 5. (b) n = 4.

Abbreviations: AVP, arginine vasopressin; EFS, electrical field stimulation; OT, oxytocin.

The V_1A_ and OT receptor antagonists, respectively, SR49059 (10^−9^–10^−7^ M) and L371257 (10^−9^–10^−7^ M), did not alter baseline muscle contractility of either stomach region but antagonized AVP and OT (Figure 3c,d) with *p*K_B_ values independent of stomach region (Table 2a). SR49059 (10^−8^ M) also antagonized OT (Figure S5) with comparable *p*A_2_ values of ~9.5 (Table 2b).

**TABLE 2:** Potency (*p*EC_50_) and tissue maximal response (E_max_) of (a) AVP and (b) OT for the increase in muscle tone, amplitude and frequency of the spontaneous contractions of human proximal and distal stomach circular muscle in the absence and presence of blockade of neurogenic, cholinergic and nitrergic activity and (a) OT receptor and (b) AVP V_1A_ receptor

(a)												
	Proximal						Distal					
	Control		+ TTX + atropine + l‐NAME		+ L371257		Control		+ TTX + atropine + l‐NAME		+ L371257	
Effect	*p*EC_50_	E_max_ (%)	*p*EC_50_	E_max_ (%)	*p*EC_50_	E_max_ (%)	*p*EC_50_	E_max_ (%)	*p*EC_50_	E_max_ (%)	*p*EC_50_	E_max_ (%)
**Tone**	9.4 ± 0.1	6.9 ± 0.2	9.1 ± 0.2	6.4 ± 0.4	9.2 ± 0.1	6.7 ± 0.2	9.4 ± 0.1	9.3 ± 0.35	8.9 ± 0.1	9.0 ± 0.3	9.2 ± 0.1	8.1 ± 0.3
**Amplitude**	9.7 ± 0.8	246.6 ± 2.8	9.1 ± 0.1	246.8 ± 2.8	9.3 ± 0.1	246.8 ± 2.8	8.5 ± 0.1	59.0 ± 1.0	8.9 ± 0.1	59.0 ± 0.9	8.9 ± 0.1	59.0 ± 0.9
**Frequency**	9.0 ± 0.2	3.0 ± 0.1	9.2 ± 0.2	3.0 ± 0.1	9.1 ± 0.1	3.0 ± 0.1	8.9 ± 0.2	3.0 ± 0.1	9.1 ± 0.1	3.2 ± 0.1	9.0 ± 0.2	3.1 ± 0.1

(b)												
	Proximal						Distal					
	Control		+ TTX + atropine + l‐NAME		+ SR49059		Control		+ TTX + atropine + l‐NAME		+ SR49059	
Effect	*p*EC_50_	E_max_ (%)	*p*EC_50_	E_max_ (%)	*p*EC_50_	E_max_ (%)	*p*EC_50_	E_max_ (%)	*p*EC_50_	E_max_ (%)	*p*EC_50_	E_max_ (%)
**Tone**	7.2 ± 0.1	4.1 ± 0.2	6.9 ± 0.8	3.9 ± 0.2	5.8 ± 0.3	3.5 ± 0.8	8.3 ± 0.1	6.1 ± 0.2	6.9 ± 0.1	6.1 ± 0.2	5.8 ± 0.1	6.1 ± 0.2
**Amplitude**	7.2 ± 0.1	141.1 ± 3.3	6.8 ± 0.1	141.1 ± 3.3	5.7 ± 0.1	141.1 ± 3.3	7.3 ± 0.1	48.6 ± 2.5	6.8 ± 2.5	48.6 ± 2.5	5.8 ± 2.5	48.6 ± 2.5
**Frequency**	7.4 ± 0.3	2.9 ± 0.1	7.2 ± 0.1	2.9 ± 0.1	5.8 ± 0.1	2.9 ± 0.1	7.7 ± 0.2	2.9 ± 0.1	7.5 ± 0.2	2.9 ± 0.1	6.1 ± 0.1	2.9 ± 0.1

*Note*: Blockade of neurogenic, cholinergic (muscarinic) and nitrergic transmission was achieved by simultaneous incubation with TTX (10^−6^ M), atropine (10^−6^ M) and l‐NAME (3 × 10^−4^ M), respectively, 30 min before examining (a) AVP and (b) OT. In separate experiments, (a) AVP and (b) OT were also investigated after blockade of OT and V_1A_ receptors using L371257 (10^−7^ M) and SR49059 (10^−7^ M), respectively. Data are mean ± SEM. n = 5.

Abbreviations: AVP, arginine vasopressin; l‐NAME, Nω‐nitro‐l‐arginine methyl ester hydrochloride; OT, oxytocin; TTX, tetrodotoxin.

#### 3.3.2 Electrically evoked contractions

**FIGURE 4:**
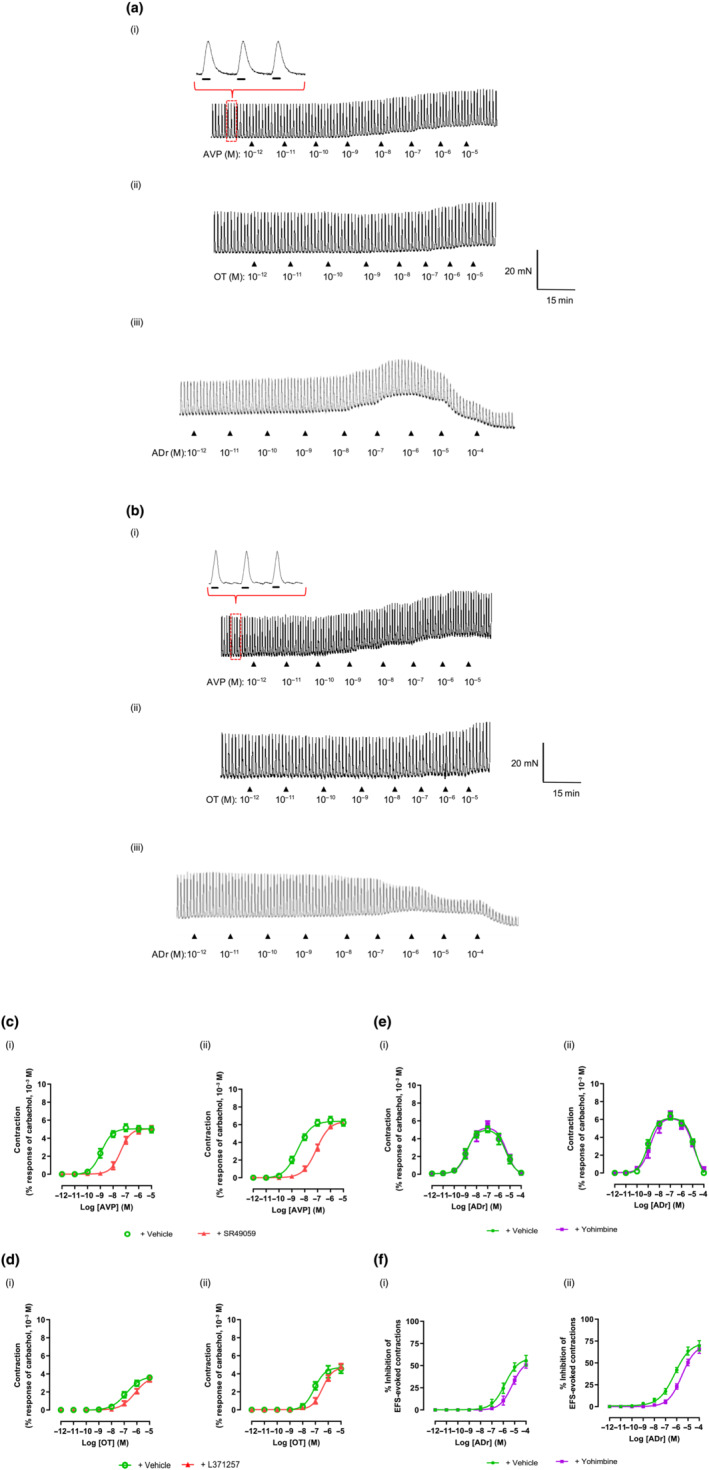
Effects of arginine vasopressin (AVP), oxytocin (OT) and adrenaline (ADr) on electrical field stimulation (EFS)‐evoked contractions of human (a) proximal and (b) distal stomach. Bars under magnified contractions indicate period of EFS. Concentration–response curves for increase in muscle tone by (c) AVP, (d) OT and (e) ADr on proximal (graphs on left) and distal (right) stomach subjected to EFS. (f) ADr‐induced inhibition of EFS‐evoked contractions of proximal (left) and distal (right) stomach. AVP, OT and ADr were applied after 30 min incubation with, respectively, SR49059 (10^−8^ M), L371257 (10^−9^ M), yohimbine (10^−7^ M) or vehicle (dimethyl sulfoxide [DMSO]/water, 0.1% v/v). Data are mean ± SEM. n = 4

EFS elicited monophasic contractions (Figure 4a,b) of similar amplitude in both stomach regions, prevented by TTX (10^−6^ M) or atropine (10^−6^ M) (Table S6).

Neither AVP nor OT affected EFS‐evoked contractions in either stomach region but increased baseline muscle tone (Figure 4a–d). SR49059 (10^−8^ M) and L371257 (10^−9^ M) were without effect on the contractions but antagonized (Figure 4c,d), respectively, AVP and OT (*p*A_2_ values consistent with *p*K_B_ values estimated on muscle strips not subjected to EFS; Table 2a,b).

### 3.4 ADr

**FIGURE 5:**
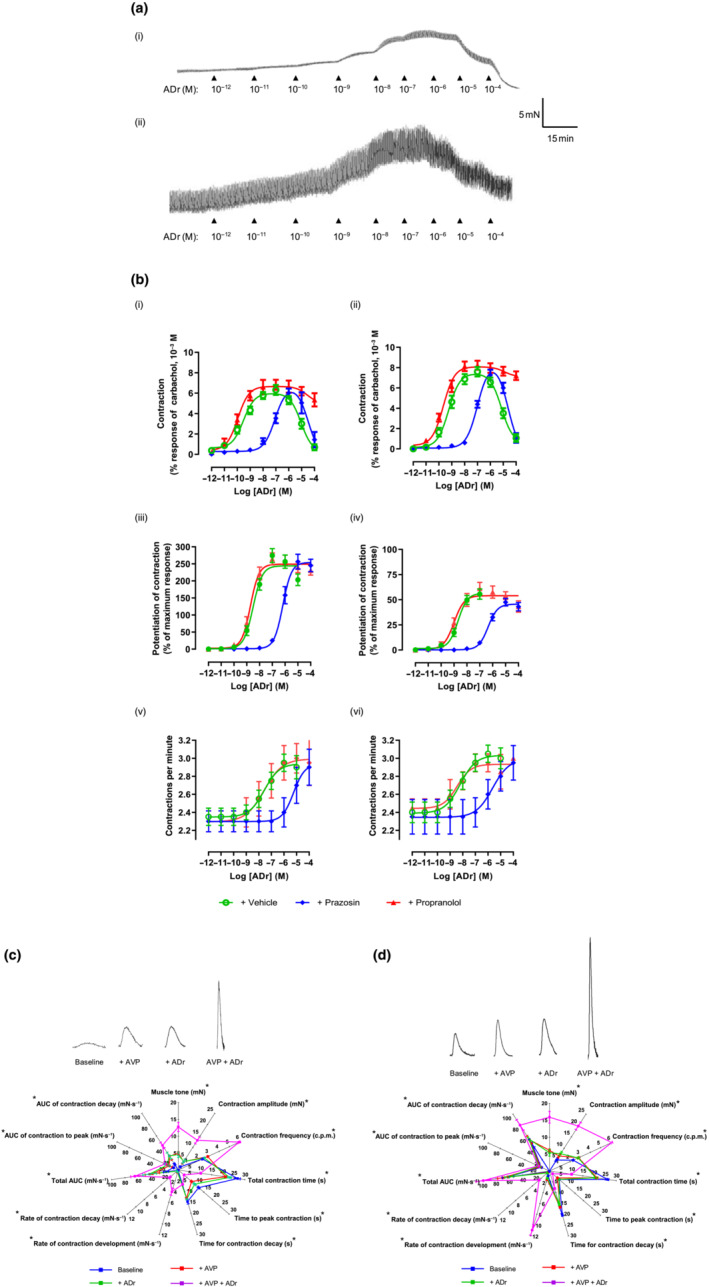
Effect of adrenaline (ADr) on (a) spontaneous contractions of human (i) proximal and (ii) distal stomach. (b) ADr‐induced increase in baseline muscle tone (i, ii), amplitude (iii, iv) and frequency (v, vi) of spontaneous contractions of proximal (graphs on left) and distal (right) stomach after 30 min incubation with prazosin (10^−7^ M), propranolol (10^−5^ M) or vehicle (dimethyl sulfoxide [DMSO]/water, 0.1% v/v). (c) Comparison of spontaneous contractions and their features in absence (baseline) and presence of [arginine vasopressin (AVP) (10^−9^ M), ADr (10^−8^ M) or co‐application of AVP (10^−9^ M) and ADr (10^−8^ M). Concentrations of both hormones approximately equivalent to their EC_75_. *P < 0.05 between data sets for AVP and ADr applied together versus sum of corresponding data for AVP and ADr applied individually. Data are mean ± SEM. n = 5

In both stomach regions, ADr produced sustained, graded increases in muscle tone up to 10^−7^ M followed by relaxation (≥10^−6^ M), accompanied by enhanced (10^−10^–10^−5^ M) or reduced (10^−4^ M) amplitude and frequency of spontaneous contractions (Figure 5). The rank order for *p*EC_50_ values (Table 1b) was, as follows: augmenting muscle tone > (2‐log_10_ units) contraction amplitude ≥ contraction frequency. ADr generated a larger maximal increase in muscle tone in distal stomach but at higher concentrations, elicited a larger, complete relaxation in proximal stomach. Prazosin (10^−7^ M) antagonized the excitatory actions of ADr, weakly reducing the muscle relaxation (Figure 5b and Table S4B). Propranolol (10^−5^ M) caused a small increase in the ability of ADr to increase muscle tone without affecting spontaneous contractions, almost abolishing the relaxations induced by high concentrations (Figure 5b). Neither prazosin nor propranolol affected spontaneous contraction activity.

ADr concentration‐dependently inhibited EFS‐evoked contractions (Figure 4a,b,f), approximately equipotently in both stomach regions (Table 1b) but ~15% more efficacious in distal stomach. Yohimbine (10^−7^ M) had no effects alone but antagonized ADr‐induced inhibition of contractions with similar *p*A_2_ values (Table S3B). Together, prazosin, propranolol and yohimbine (10^−7^ M each) abolished all actions of ADr.

### 3.5 AVP and ADr

To investigate whether AVP and ADr could interact functionally, they were applied at concentrations equivalent to that producing 75% of their maximum: 10^−9^ and 10^−8^ M, respectively. Co‐administration caused synergistic augmentation of muscle tone and amplitude and frequency of spontaneous contractions, compared with when applied individually (Figure 5c,d). There was no difference in pattern of synergy between AVP and ADr in the two stomach regions.

### 3.6 Receptor expression

**FIGURE 6:**
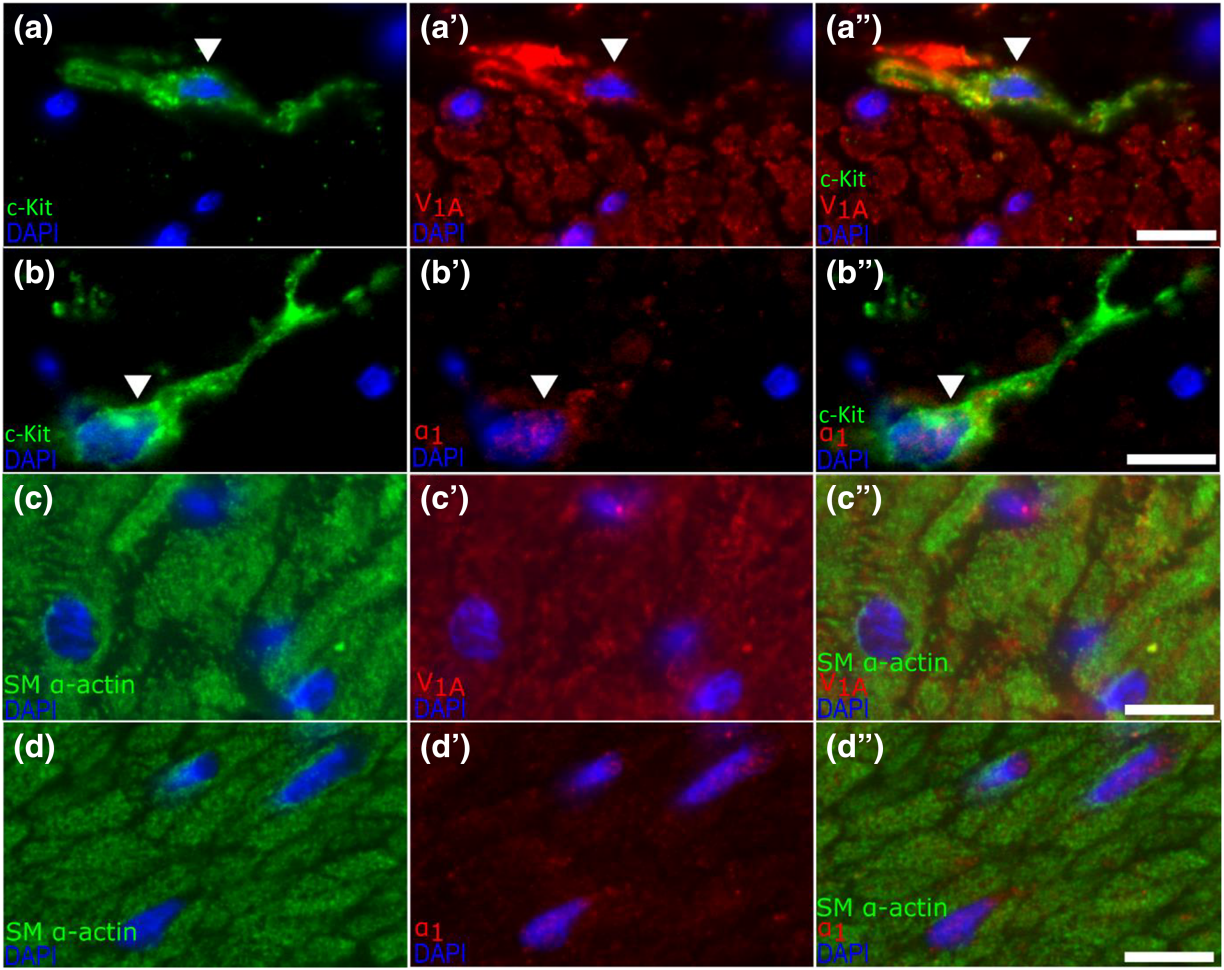
Fluorescence staining of c‐Kit and V_1A_ or α_1_ in human distal stomach circular muscle. White arrows across the panels indicate the same cell. Panel (a) shows a cell with 4′,6‐diamidino‐2‐phenylindole (DAPI)‐stained nucleus and emanating c‐Kit‐positive extensions, indictive of an interstitial cell of Cajal (ICC) (white arrow). The same cell is shown (a′) to stain for V_1A_, and again in the merged images (a″). Panel (b) shows a DAPI‐stained cell (white arrow) with c‐Kit‐positive extensions, (b′) shows the same cell staining for α_1_, whereas (b″) merges these images. Panel (c) shows a representative area of muscle with SM α‐actin‐positive muscle cells, (c′) shows these same cells staining for V_1A_ and (c″) merges these images. Panel (d) is arranged similarly and shows muscle cells staining for α_1_. All images were captured at 40× magnification using Olympus BX61 microscope and SmartCapture3 software. The white scale bar represents 10 μm

In preliminary studies, ICCs (DAPI‐stained nucleus with c‐Kit‐positive irregular processes emanating from cell bodies) were not stained by mast cell tryptase, further distinguishing these cells from the rounder mast cells, both c‐Kit positive (Figure S7). V_1A_ receptor and α_1_‐adrenoceptor immunofluorescence was detected on, respectively, 69.4 ± 12.5% and 61.1 ± 3.5% (mean of three ICCs in circular muscle from each of six donors; Figure 6). These data were not influenced by diagnosis of diabetes mellitus or inter‐observer differences (Figure S8). Smooth muscle cells were identified by a DAPI‐stained nucleus and αSMA‐positive staining. Each of three smooth muscle cells from each of six donors showed positive immunofluorescence for V_1A_ receptors, with 44.5.1 ± 18.1% immunofluorescent for α_1_‐adrenoceptors.

## 4 DISCUSSION

Proximal and distal human stomach spontaneously and rhythmically contracted at ~3 c.p.m., independent of donor sex or diabetes mellitus. The contractions were ‘myogenic’ (unaffected by blocking neuronal functions) and likely initiated by spontaneous ICC depolarization (Rhee et al., 2011). Others, measuring electrical and sometimes mechanical activity of human stomach, reported higher frequencies (fundus, corpus, antrum: 4.2, 3.9, 4.9 c.p.m.; fundus, corpus: 5–6; antrum: 4–7; El‐Sharkawy et al., 1978; Rhee et al., 2011; Sinn et al., 2010). The reasons for the differences are unclear. Perhaps pinning/impalement with microelectrodes caused tissue damage (O'Grady et al., 2012). A less likely possibility is biological variation between patients with different diagnoses, since others found similar gastric electrical activity (~3 c.p.m.) in different GI disorders (Hinder & Kelly, 1977).

Myogenic contraction frequency was within the ranges recorded by EGG in healthy volunteers (2–4 c.p.m.; Yin & Chen, 2013), showed no region dependence and appeared similar to slow wave electrical frequencies in the same areas of anaesthetized, fasted humans (O'Grady et al., 2010). Thus, it seems unlikely that the present data are greatly skewed by studying tissue from obese individuals. Others have suggested the existence of a frequency gradient of electrical activity from corpus to antrum (Hinder & Kelly, 1977), although robust measurements of muscle contraction frequency have yet to be obtained in vivo. Furthermore, it remains possible that gastric electrical or myogenic activity in vivo can be influenced by substances released from the mucosa (e.g., Cai et al., 2022), removed in the present experiments. Notably, the myogenic contractions in vitro were 5× larger in distal, compared with proximal, stomach, reaching maximum tension more quickly and returning to baseline more slowly. Since smooth muscle cells and ICCs (together with platelet‐derived growth factor receptor‐α^+^ cells) exist as interactive electrically coupled syncytia (Sanders et al., 2014), these contraction parameters might be expected to reflect the rates of rise and amplitude of electrical slow waves. Indeed, similar characteristics were recorded by high‐resolution mapping in stomach of patients undergoing surgery (O'Grady et al., 2010). Region‐dependent differences could reflect different resting membrane potentials (Rhee et al., 2011) and the need for distal stomach to generate stronger contractions following meals (Pal et al., 2004).

Low concentrations of AVP caused muscle contraction and stimulated myogenic contractions. This was independent of neuronal activity or mast cell functions (stimulated by AVP activating enteric neurons in mice with 2,4,6‐trinitrobenzenesulfonic acid [TNBS]‐induced colitis; Dou et al., 2019), and AVP did not modulate cholinergically mediated contractions. In proximal stomach, the AVP‐induced increase in tone was maintained and appeared unrelated to increases in spontaneous contractions. The likely consequences in vivo would be reduced proximal stomach storage capacity, ‘pushing’ contents to the antrum, particularly liquids (Indireshkumar et al., 2000). In distal stomach, the smaller increases in muscle tone appeared to track the increase in spontaneous contraction activity. In both regions, AVP increased amplitude, rate and frequency of myogenic contractions, especially in distal stomach. These actions were mediated through V_1A_, not OT, receptors, independently of neuronal activity. This conclusion finds consistency with V_1A_ receptor mRNA in human stomach neuromuscular tissue (extending previous findings for full‐thickness human stomach biopsies; Monstein et al., 2008).

The effects of AVP occurred at concentrations measured in human blood plasma during nausea (Table S7). For example, AVP concentrations were 8.2 × 10^−10^ M following apomorphine (near EC_50_ in the present study). In other investigations, infusing AVP induced nausea and EGG changes at blood levels of 3.9–6.4 × 10^−10^ M, concentrations which cause stomach contraction. Notably, OT was less effective than AVP, although both OT and V_1A_ receptors were activated by OT. These observations, combined with the absence of significant increases in plasma OT during nausea in humans (see Introduction), are consistent with the view that OT does not induce nausea. In healthy volunteers, OT did not affect gastric emptying, although satiety scores were reduced (Borg et al., 2011).

The use of single concentrations of AVP showed that the effects on gastric motility plateaued in a concentration‐dependent manner over 2–10 min, consistent with the onset of nausea within 5 min of AVP infusion (Caras et al., 1997).

To address the question, ‘Could AVP activity combine with other substances released during nausea?’, the actions of ADr were studied. Immediate, sustained increases in plasma ADr occur during motion sickness (~5–15 × 10^−11^ g·ml^−1^) and tilt‐induced syncope, when ADr and AVP are released (Table S7). Further, ADr infusions disrupt human gastric slow waves (Lien et al., 2003). In the present study, low ADr concentrations caused muscle contraction and increased amplitude and rate of myogenic contractions in both stomach regions (via α_1_‐adrenoceptor activation), whereas higher concentrations relaxed the muscle (β‐adrenoceptor activation). ADr reduced EFS‐evoked, cholinergically mediated contractions via α_2_‐adrenoceptor activation. Surprisingly, these data represent the first complete analysis for human stomach. Others showed ADr evoked muscle relaxation in human corpus/antrum (Bennett & Whitney, 1966), contractions in some stomach and pylorus muscle strips and biphasic responses or inhibition in others (Haffner et al., 1969). In human internal anal sphincter, α_1A_‐adrenoceptor activation caused muscle contraction (Owaki et al., 2015).

Co‐administration of submaximally effective concentrations of AVP and ADr augmented muscle tone, contraction amplitude, rate and frequency of spontaneous contractions in a synergistic manner, compared with individual applications. Notably, the increased antrum contraction frequency caused by AVP and ADr together falls within the EGG tachygastria range recorded in subjects reporting nausea during AVP infusion (Kim et al., 1997) and vection (Koch et al., 1990; Lien et al., 2003; Xu et al., 1993). A pathway by which AVP and ADr might interact is suggested by the localization of V_1A_ receptor and α_1_‐adrenoceptor immunofluorescence on ICCs and muscle cells. These experiments were challenging because of tissue autofluorescence, possible loss of epitopes with degradation during tissue processing and use of paraffin‐embedded sections, which sometimes made it difficult to see the full extent of ICC extensions with nuclei. Nevertheless, co‐expression of the receptors with muscle was consistent with the functional data and co‐expression with both muscle and ICCs suggests mechanisms of synergy that involve intracellular pathways. These pathways are unknown, although vascular studies demonstrated functional synergy between AVP and noradrenaline involving Ca^2+^ channel activation (Noguera et al., 1997) or prostaglandin release (Karmazyn et al., 1978). Interestingly, only an insignificant expression of V_1A_ and α_1_‐adrenoceptor receptor mRNA occurred in ICCs from the mouse intestine (Lee et al., 2017), suggesting species differences in receptor distribution, consistent with release of OT instead of AVP by rodents following emetogenic stimuli (Horn et al., 2013).

The ability of AVP and ADr (especially together) to increase myogenic contractions is consistent with AVP‐induced EGG tachygastria (Caras et al., 1997), a role for catecholamines in inducing tachygastria during motion sickness (Chen et al., 1993) and with electrical tachygastria during nausea (Koch, 1997). However, they differ from bradygastria reported in response to AVP (Kim et al., 1997) (perhaps because of different recording methods and concentrations of AVP; Koch, 1997), with antral hypomotility and reduced gastric contraction amplitude associated with the electrical tachygastria/dysrhythmia (Faas et al., 2001; Lien et al., 2003; Ouyang et al., 2005), and with delayed emptying during nausea (Faas et al., 2001; Koch, 2014). These mismatches between the different data are difficult to dismiss, given the robustness of the present results in vitro. Perhaps the large increase in rate, frequency and amplitude of contractions of distal stomach by AVP and ADr stimulates vagal nerve mechanoreceptors, resulting in vago‐vagal reflex inhibition of gastric motility. Additionally, the use of muscle strips limits functional interpretation. For example, we do not know (i) if the large, high‐frequency contractions in vitro are propulsive; (ii) if the larger increase in contraction parameters in distal stomach causes dysrhythmia by destroying entrainment with the more slowly developing smaller contractions originating in the corpus (as in mice; Kim et al., 2003); (iii) if the changes occur evenly across the distal stomach (in dogs, intravenous AVP caused ectopic dysrhythmia; Du et al., 2016); and (iv) if the contractions operate against a closed pyloric sphincter (which itself may be affected by AVP/ADr), resulting in delayed gastric emptying.

## 5 CONCLUSIONS

The present experiments demonstrate region‐dependent differences in human stomach rhythmic spontaneous myogenic contractions, generating greater maximum muscle tension and occurring at a faster rate in distal stomach. Marked stimulation by AVP occurred at low concentrations, within the range reported in human plasma during nausea and vomiting. Pathophysiological relevance was re‐enforced by synergistic activity between AVP and ADr, reflecting the known pattern of co‐release during nausea. ADr also decreased cholinergic transmission to the muscle. These observations support the suggestion that multiple stimuli act together, reaching a ‘tipping point’ at which central pathways generate nausea (Farmer et al., 2015; LaCount et al., 2011; Napadow et al., 2013; Stern et al., 2011). Interestingly, this could mean that blocking the gastric actions of either AVP or ADr alone may not fully abolish nausea. Thus, following illusory‐self motion, the α_1_‐adrenoceptor antagonist phentolamine delayed the time to reach maximum tachygastria and reduced its magnitude and reduced but did not abolish nausea (Hasler et al., 1995).

Studies are now needed to understand exactly how the combined activity of AVP and ADr delays gastric emptying and causes nausea. Nevertheless, the proposed pathway challenges the long‐held assumption that ‘nauseagenic hormones’ necessarily induce nausea solely via the AP (Borison, 1989).

## CONFLICTS OF INTEREST

GJS was in receipt of research funding from Takeda Pharmaceuticals and acts as scientific advisor for BYOMass. GO'G and AG are affiliated with Alimetry Ltd. Other authors do not have competing interests.

## AUTHOR CONTRIBUTIONS

RM and EC performed the studies and initial data analysis, planned by RM, EC, AP and GJS. MS was involved in the data collection. JL, KD and EC enabled access to the human tissue and patient demographics. AG wrote the software enabling analysis of the parameters of contraction, visualized by RM. RM and GJS wrote the first draft of the paper, critiqued by GO'G and PLRA with the final draft agreed by all authors. GJS obtained the funding.

## DECLARATION OF TRANSPARENCY AND SCIENTIFIC RIGOUR

This Declaration acknowledges that this paper adheres to the principles for transparent reporting and scientific rigour of preclinical research as stated in the *BJP* guidelines for Design & Analysis, and Animal Experimentation, and as recommended by funding agencies, publishers and other organizations engaged with supporting research.

## Supporting information


**Figure S1:** Comparison between Glyceraldehyde 3‐phosphate dehydrogenase (GAPDH) mRNA levels in human proximal and distal stomach muscle. (B) Expression of AVP, OT and ADr receptor mRNA in human proximal (P) and distal (D) stomach muscle normalised to GAPDH mRNA levels. Ct is inversely proportional to level of gene expression. Data are mean ± S.E.M.; n = 5–8.
**Figure S2:** Comparison between the various features of the spontaneous contraction waveform of male (n = 7) and female (n = 37) human (A) proximal and (B) distal stomach circular muscle, and of (C) proximal and (D) distal stomach circular muscle from people with (n = 9) and without (n = 32) diabetes (n = 2 patient records unavailable). Also shown are the maximal increases in muscle tone caused by carbachol (10^−3^ M). Each value represents the mean ± S.E.M. Total n = 44 donors.
**Figure S3:** Comparison between the various features of the spontaneous contraction waveform and baseline muscle tone of human (A) proximal and (B) distal stomach circular muscle before (Baseline) and after 30 min incubation with atropine (10^−6^ M). Each value represents the mean ± S.E.M. n = 6.
**Figure S4:** Spontaneous contractions of human (A) proximal and (B) distal stomach circular muscle in the absence and presence of a single challenge of individual strips with AVP at the concentration indicated. (C) Comparison of the AVP‐induced increase in muscle tone (I,ii), and the amplitude (iii,iv) and frequency (v,vii) of spontaneous contractions of human proximal (graphs on the left) and distal (graphs on the right) stomach by single or cumulative application of AVP. (D) Time course profile for the AVP‐induced increase in muscle tone (I,ii), and the amplitude (iii,iv) and frequency (v,vii) of spontaneous contractions of human proximal (graphs on the left) and distal (graphs on the right) stomach. Data are mean ± S.E.M.; n = 4.
**Figure S5:** (A) AVP‐ and (B) OT‐induced increase in muscle tone (i, ii) and the amplitude (iii, iv) and frequency (v, vi) of spontaneous contractions of human proximal (graphs on the left) and distal (graphs on the right) stomach, after a 30 min incubation with either TTX (10^−6^ M), atropine (10^−6^ M), a combination of tetrodotoxin (10^−6^ M), atropine (10^−6^ M) and L‐NAME (3 x 10^−4^ M), SR49059 (10^−8^ M) or L371257 (10^−7^ M) or their vehicle (DMSO and water, 0.5% v/v). Data are mean ± S.E.M.; n = 5.
**Figure S6:** AVP‐induced increase in muscle tone (i, ii) and amplitude (iii, iv) and frequency (v, vi) of spontaneous contractions of human proximal (graphs on left) and distal (right) stomach, after 30 min incubation with mepyramine (10^−7^ M), cromolyn (10^−5^ M) or their vehicle (water 0.1% v/v). Data are mean ± S.E.M.; n = 4.
**Figure S7:** (A) Representative mast cell staining in distal stomach muscle. Image A: Mast cell was counterstained for 4′,6‐diamidino‐2‐phenylindole (DAPI) (white arrow). Image A′ and A″ show the cytoplasmic localisation of the mast cell tryptase (MCT) stain (A″ includes DAPI stain). Images were captured at 40x magnification The scale bar 5 μm. (B) Distinguishing between mast cells and interstitial cells of Cajal (ICC) in distal stomach muscle. A: DAPI counterstained cells. Images A′ and A″ highlight a mast cell (yellow arrow) staining for respectively, c‐KIT, and mast cell tryptase (MCT). Image A‴ shows the merged image together with an ICC (white arrow) staining for c‐KIT; extensions, when present, were only seen protruding from the ICC whereas mast cells often appeared with ‘rounder’ nuclei. Images were captured at 20x magnification The white scale bar is 10 μm. All images were taken using an Olympus BX61 microscope and SmartCapture3 software. MCT antibody used for preliminary studies was Agilent Dako (M705229‐2), c‐KIT antibody used was Agilent Dako (A4502). ICC and Mast cell differentiation criteria was confirmed for multiple cells in separate images from 4 different donors using the same primary antibodies whilst optimising other staining conditions.
**Figure S8:** Bland–Altman (average vs difference) analyses showing lack of inter‐rater bias between two investigators observing immunofluorescence for the V1A receptor on (A) c‐Kit and (B) α‐SMA ‐positive cells and α1 adrenoceptor on (C) c‐Kit and (D) α‐SMA ‐positive cells. c‐Kit and α‐SMA positive cells indicated ICCs and myocytes, respectively. Analyses was performed on immunofluorescence images of three randomly selected cells of both cell types in the distal stomach muscle layer of 6 people. Dotted lines above and below origin (0) are 95% confidence intervals.

**Data S1** Supporting information

**Table S1:** Anthropometric measurements and medical history of people donating stomach tissue.
**Table S2**: (A) Potency (*p*EC_50_) and tissue maximal effect (E_max_) of AVP for the increase in baseline muscle tension in the absence and presence of electrical field stimulation (EFS), and the amplitude and frequency of the spontaneous contractions of human proximal and distal stomach circular muscle when used fresh after resection or overnight (15–18 h) storage at 4°C in Krebs–Henseleit solution. (B) Amplitude of the EFS‐evoked contractions of fresh and overnight stored muscle strips before the application of AVP.**Table S3:** Potency (*p*EC_50_) and tissue maximal response (E_max_) of AVP for the increase in baseline muscle tone, amplitude and frequency of the spontaneous contractions of human proximal and distal stomach circular muscle obtained from concentration‐response curves constructed using a cumulative and single dose per muscle strip dosing technique.
**Table S4.** (A) Schild slopes and *p*K_B_ values of SR49059 and L371257 for antagonism of respectively, AVP‐ and OT‐induced increase in muscle tone, amplitude and frequency of spontaneous contractions of human proximal and distal stomach circular muscle. (B) *p*A_2_ values of prazosin and yohimbine for the antagonism of the ADr‐induced changes in muscle tone and spontaneous contraction activity and inhibition of EFS‐evoked contractions of human proximal and distal stomach circular muscle.
**Table S5:** Potency (*p*EC_50_) and tissue maximal response (E_max_) of AVP for the increase in muscle tone, amplitude and frequency of the spontaneous contractions of human proximal and distal stomach circular muscle in the absence and presence of blockade of histamine H_1_ receptor and stabilisation of mast cells.
**Table S6.** Amplitude of the electrical field stimulation (EFS)‐evoked contractions of human isolated proximal and distal stomach and when expressed as a fraction of the maximal contraction to carbachol (10^−3^ M), and the inhibitory effect of TTX (10^−6^ M) and atropine (10^−6^ M).
**Table S7:** Baseline and raised plasma concentrations of AVP and ADr in response to stimuli evoking nausea in human subjects

## Data Availability

All data pertinent to this work are contained within the manuscript. The data that support the findings of this study are available from the corresponding author upon reasonable request. Some data may not be made available because of privacy or ethical restrictions. SAGER Guidelines have been followed.

## References

[bph15943-bib-0001] Alexander, S. P. H. , Christopoulos, A. , Davenport, A. P. , Kelly, E. , Mathie, A. , Peters, J. A. , Veale, E. L. , Armstrong, J. F. , Faccenda, E. , Harding, S. D. , Pawson, A. J. , Southan, C. , Davies, J. A. , Abbracchio, M. P. , Alexander, W. , Al‐Hosaini, K. , Bäck, M. , Barnes, N. M. , Bathgate, R. , … Ye, R. D. (2021). The Concise Guide to PHARMACOLOGY 2021/22: G protein‐coupled receptors. British Journal of Pharmacology, 178, S27–S156. 34529832 10.1111/bph.15538

[bph15943-bib-0062] Alexander, S. P. H. , Roberts, R. E. , Broughton, B. R. S. , Sobey, C. G. , George, C. H. , Stanford, S. C. , Cirino, G. , Docherty, J. R. , Giembycz, M. A. , Hoyer, D. , Insel, P. A. , Izzo, A. A. , Ji, Y. , MacEwan, D. J. , Mangum, J. , Wonnacott, S. , & Ahluwalia, A. (2018). Goals and practicalities of immunoblotting and immunohistochemistry: A guide for submission to the British Journal of Pharmacology. British Journal of Pharmacology, 175, 407–411. 10.1111/bph.14112 29350411 PMC5773976

[bph15943-bib-0002] Bennett, A. , & Whitney, B. (1966). A pharmacological investigation of human isolated stomach. British Journal of Pharmacology and Chemotherapy, 27, 286–298. 10.1111/j.1476-5381.1966.tb01663.x 4381775 PMC1510827

[bph15943-bib-0003] Bichet, D. , Bouvier, M. , Chini, B. , Gimpl, G. , Guillon, G. , Kimura, T. , Knepper, M. , Lolait, S. , Manning, M. , Mouillac, B. , O'Carroll, A. M. , Serradeil‐Le Gal, C. , Soloff, M. , Verbalis, J. G. , Wheatley, M. , & Zingg, H. H. (2019). Vasopressin and oxytocin receptors (version 2019.4) in the IUPHAR/BPS Guide to Pharmacology Database. IUPHAR/BPS Guide to Pharmacology CITE, 2019(4). 10.2218/gtopdb/F66/2019.4

[bph15943-bib-0004] Borg, J. , Simrén, M. , & Ohlsson, B. (2011). Oxytocin reduces satiety scores without affecting the volume of nutrient intake or gastric emptying rate in healthy subjects. Neurogastroenterology and Motility, 23, 56–61. 10.1111/j.1365-2982.2010.01599.x 20868426

[bph15943-bib-0005] Borison, H. L. (1989). Area postrema: Chemoreceptor circumventricular organ of the medulla oblongata. Progress in Neurobiology, 32, 351–390. 10.1016/0301-0082(89)90028-2 2660187

[bph15943-bib-0006] Broad, J. , Mukherjee, S. , Samadi, M. , Martin, J. E. , Dukes, G. E. , & Sanger, G. J. (2012). Regional‐ and agonist‐dependent facilitation of human neurogastrointestinal functions by motilin receptor agonists. British Journal of Pharmacology, 167, 763–774. 10.1111/j.1476-5381.2012.02009.x 22537158 PMC3575777

[bph15943-bib-0007] Cai, W. , Makwana, R. , Straface, M. , Gharibans, A. , Andrews, P. L. R. , & Sanger, G. J. (2022). Evidence for tetrodotoxin‐resistant spontaneous myogenic contractions of mouse isolated stomach that are dependent on acetylcholine. British Journal of Pharmacology, 179(6), 1187–1200. 10.1111/bph.15685 34519057 PMC9297954

[bph15943-bib-0008] Camilleri, M. , Chedid, V. , Ford, A. C. , Haruma, K. , Horowitz, M. , Jones, K. L. , Low, P. A. , Park, S.‐Y. , Parkman, H. P. , & Stanghellini, V. (2018). Gastroparesis. Nature Reviews. Disease Primers, 4, 41. 10.1038/s41572-018-0038-z 30385743

[bph15943-bib-0009] Caras, S. D. , Soykan, I. , Beverly, V. , Lin, Z. , & McCallum, R. W. (1997). The effect of intravenous vasopressin on gastric myoelectrical activity in human subjects. Neurogastroenterology and Motility, 9, 151–156. 10.1046/j.1365-2982.1997.d01-37.x 9347470

[bph15943-bib-0010] Carpenter, D. O. , Briggs, D. B. , Knox, A. P. , & Strominger, N. (1988). Excitation of area postrema neurons by transmitters, peptides, and cyclic nucleotides. Journal of Neurophysiology, 59, 358–369. 10.1152/jn.1988.59.2.358 2895167

[bph15943-bib-0011] Chen, J. , McCallum, R. W. , & Richards, R. (1993). Frequency components of the electrogastrogram and their correlations with gastrointestinal contractions in humans. Medical & Biological Engineering & Computing, 31, 60–67. 8326766 10.1007/BF02446895

[bph15943-bib-0012] Curtis, M. J. , Alexander, S. , Cirino, G. , Docherty, J. R. , George, C. H. , Giembycz, M. A. , Hoyer, D. , Insel, P. A. , Izzo, A. A. , Ji, Y. , MacEwan, D. J. , Sobey, C. G. , Stanford, S. C. , Teixeira, M. M. , Wonnacott, S. , & Ahluwalia, A. (2018). Experimental design and analysis and their reporting II: Updated and simplified guidance for authors and peer reviewers. British Journal of Pharmacology, 175, 987–993. 10.1111/bph.14153 29520785 PMC5843711

[bph15943-bib-0013] Dou, D. , Chen, L. , Di, H. , Song, Z. , Li, S. , Bu, X. , Dai, Q. , Wang, S. , Li, J. X. , Zhu, X. , & Jing, H. (2019). Vasopressin augments TNBS‐induced colitis through enteric neuronal V_1a_ receptor‐mediated COX‐2‐dependent prostaglandin release from mast cells in mice. Neurogastroenterology and Motility, 31, e13493. 10.1111/nmo.13493 30334342

[bph15943-bib-0014] Du, P. , O'Grady, G. , Paskaranandavadivel, N. , Tang, S.‐J. , Abell, T. , & Cheng, L. K. (2016). Simultaneous anterior and posterior serosal mapping of gastric slow‐wave dysrhythmias induced by vasopressin. Experimental Physiology, 101(9), 1206–1217. 10.1113/EP085697 27265885 PMC5140776

[bph15943-bib-0015] Edwards, C. M. , Carmichael, J. , Baylis, P. H. , & Harris, A. L. (1989). Arginine vasopressin—A mediator of chemotherapy induced emesis? British Journal of Cancer, 59, 467–470. 10.1038/bjc.1989.96 2930716 PMC2247086

[bph15943-bib-0016] El‐Sharkawy, T. Y. , Morgan, K. G. , & Szurszewski, J. H. (1978). Intracellular electrical activity of canine and human gastric smooth muscle. The Journal of Physiology, 279, 291–307. 10.1113/jphysiol.1978.sp012345 671352 PMC1282616

[bph15943-bib-0017] Faas, H. , Feinle, C. , Enck, P. , Grundy, D. , & Boesiger, P. (2001). Modulation of gastric motor activity by a centrally acting stimulus, circular vection, in humans. The American Journal of Physiology, 280, G850–G857. 10.1152/ajpgi.2001.280.5.G850 11292592

[bph15943-bib-0018] Farmer, A. D. , Ban, V. F. , Coen, S. J. , Sanger, G. J. , Barker, G. J. , Gresty, M. A. , Giampietro, V. P. , Williams, S. C. , Webb, D. L. , Hellström, P. M. , Andrews, P. L. R. , & Aziz, Q. (2015). Visually induced nausea causes characteristic changes in cerebral, autonomic and endocrine function in humans. The Journal of Physiology, 593, 1183–1196. 10.1113/jphysiol.2014.284240 25557265 PMC4358679

[bph15943-bib-0019] Griffante, C. , Green, A. , Curcuruto, O. , Haslam, C. P. , Dickinson, B. A. , & Arban, R. (2005). Selectivity of d[Cha^4^]AVP and SSR149415 at human vasopressin and oxytocin receptors: Evidence that SSR149415 is a mixed vasopressin V_1b_/oxytocin receptor antagonist. British Journal of Pharmacology, 146, 744–751. 10.1038/sj.bjp.0706383 16158071 PMC1751202

[bph15943-bib-0020] Haffner, J. F. W. , Liavåg, I. , & Setekleiv, J. (1969). Excitatory adrenergic receptors in the human stomach and pylorus. Scandinavian Journal of Gastroenterology, 4, 145–150. 10.3109/00365526909179922 4390626

[bph15943-bib-0021] Hasler, W. L. , Kim, M. S. , Chey, W. D. , Stevenson, V. , Stein, B. , & Owyang, C. (1995). Central cholinergic and alpha‐adrenergic mediation of gastric slow wave dysrhythmias evoked during motion sickness. The American Journal of Physiology, 268, G539–G547. 7733281 10.1152/ajpgi.1995.268.4.G539

[bph15943-bib-0022] Heyer, G. L. , Boles, L. H. , Harvey, R. A. , & Cismowski, M. J. (2017). Gastric myoelectrical and neurohormonal changes associated with nausea during tilt‐induced syncope. Neurogastroenterology and Motility, e13220. 10.1111/nmo.13220 28960795

[bph15943-bib-0023] Hinder, R. A. , & Kelly, K. A. (1977). Human gastric pacesetter potential. Site of origin, spread, and response to gastric transaction and proximal gastric vagotomy. American Journal of Surgery, 133, 29–33. 10.1016/0002-9610(77)90187-8 835775

[bph15943-bib-0024] Holt, N. F. , & Haspel, K. L. (2010). Vasopressin: A review of therapeutic applications. Journal of Cardiothoracic and Vascular Anesthesia, 24, 330–347. 10.1053/j.jvca.2009.09.006 19945299

[bph15943-bib-0025] Horn, C. C. , Kimball, B. A. , Wang, H. , Kaus, J. , Dienel, S. , Nagy, A. , Gathright, G. R. , Yates, B. J. , & Andrews, P. L. R. (2013). Why can't rodents vomit? A comparative behavioral, anatomical, and physiological study. PLoS ONE, 8, e60537. 10.1371/journal.pone.0060537 23593236 PMC3622671

[bph15943-bib-0026] Indireshkumar, K. , Brasseur, J. G. , Faas, H. , Hebbard, G. S. , Kunz, P. , Dent, J. , Feinle, C. , Li, M. , Boesiger, P. , Fried, M. , & Schwizer, W. (2000). Relative contributions of “pressure pump” and “peristaltic pump” to gastric emptying. The American Journal of Physiology, 278, G604–G616. 10.1152/ajpgi.2000.278.4.G604 10762615

[bph15943-bib-0027] Karmazyn, M. , Manky, M. S. , & Horrobin, D. F. (1978). Changes of vascular reactivity induced by low vasopressin concentrations: Interactions with cortisol and lithium and possible involvement of prostaglandins. Endocrinology, 1978(102), 1230–1236. 10.1210/endo-102-4-1230 744021

[bph15943-bib-0028] Kim, M. S. , Chey, W. D. , Owyang, C. , & Hasler, W. L. (1997). Role of plasma vasopressin as a mediator of nausea and gastric slow wave dysrhythmias in motion sickness. The American Journal of Physiology, 272, G853–G862. 9142918 10.1152/ajpgi.1997.272.4.G853

[bph15943-bib-0029] Kim, T. W. , Koh, S. D. , Ordög, T. , Ward, S. M. , & Sanders, K. M. (2003). Muscarinic regulation of pacemaker frequency in murine gastric interstitial cells of Cajal. The Journal of Physiology, 546, 415–425. 10.1113/jphysiol.2002.028977 12527728 PMC2342515

[bph15943-bib-0030] Koch, K. L. (1997). A noxious trio: Nausea, gastric dysrhythmias and vasopressin. Neurogastroenterology and Motility, 9, 141–142. 10.1046/j.1365-2982.1997.d01-44.x 9347468

[bph15943-bib-0031] Koch, K. L. (2014). Gastric dysrhythmias: A potential objective measure of nausea. Experimental Brain Research, 232, 2553–2561. 10.1007/s00221-014-4007-9 24916149

[bph15943-bib-0032] Koch, K. L. , Stern, R. M. , Vasey, M. W. , Seaton, J. F. , Demers, L. M. , & Harrison, T. S. (1990). Neuroendocrine and gastric myoelectrical responses to illusory self‐motion in humans. The American Journal of Physiology, 258, E304–E310. 2137678 10.1152/ajpendo.1990.258.2.E304

[bph15943-bib-0033] LaCount, L. T. , Barbieri, R. , Park, K. , Kim, J. , Brown, E. N. , Kuo, B. , & Napadow, V. (2011). Static and dynamic autonomic response with increasing nausea perception. Aviation, Space, and Environmental Medicine, 82, 424–433. 21485400 10.3357/asem.2932.2011 PMC3137518

[bph15943-bib-0034] Lee, M. Y. , Ha, S. E. , Park, C. , Park, P. J. , Fuchs, R. , Wei, L. , Jorgensen, B. G. , Redelman, D. , Ward, S. M. , Sanders, K. M. , & Ro, S. (2017). Transcriptome of interstitial cells of Cajal reveals unique and selective gene signatures. PLoS ONE, 12, e0176031. 10.1371/journal.pone.0176031 28426719 PMC5398589

[bph15943-bib-0035] Lien, H. C. , Sun, W. M. , Chen, Y. H. , Kim, H. , Hasler, W. , & Owyang, C. (2003). Effects of ginger on motion sickness and gastric slow‐wave dysrhythmias induced by circular vection. The American Journal of Physiology, 284, G481–G489. 10.1152/ajpgi.00164.2002 12576305

[bph15943-bib-0036] Makwana, R. , Loy, J. , Adebibe, M. , Devalia, K. , Andrews, P. L. R. , & Sanger, G. J. (2021). Copeptin, a surrogate marker of arginine^8^ vasopressin, has no ability to modulate human and mouse gastric motility. European Journal of Pharmacology, 892, 173740. 10.1016/j.ejphar.2020.173740 33220268

[bph15943-bib-0037] Monstein, H. J. , Truedsson, M. , Ryberg, A. , & Ohlsson, B. (2008). Vasopressin receptor mRNA expression in the human gastrointestinal tract. European Surgical Research, 40, 34–40. 10.1159/000108655 17890865

[bph15943-bib-0038] Napadow, V. , Sheehan, J. D. , Kim, J. , Lacount, L. T. , Park, K. , Kaptchuk, T. J. , Rosen, B. R. , & Kuo, B. (2013). The brain circuitry underlying the temporal evolution of nausea in humans. Cerebral Cortex, 23, 806–813. 10.1093/cercor/bhs073 22473843 PMC3593575

[bph15943-bib-0039] Noguera, I. , Medina, P. , Segarra, G. , Martínez, M. C. , Aldasoro, M. , Vila, J. M. , & Lluch, S. (1997). Potentiation by vasopressin of adrenergic vasoconstriction in the rat isolated mesenteric artery. British Journal of Pharmacology, 122, 431–438. 10.1038/sj.bjp.0701397 9351498 PMC1564956

[bph15943-bib-0040] O'Grady, G. , Du, P. , Cheng, L. K. , Egbuji, J. U. , Lammers, W. J. E. P. , Windsor, J. A. , & Pullan, A. J. (2010). Origin and propagation of human gastric slow wave activity defined by high‐resolution mapping. The American Journal of Physiology, 299, G585–G592. 10.1152/ajpgi.00125.2010 PMC2950696 20595620

[bph15943-bib-0041] O'Grady, G. , Pullan, A. J. , & Cheng, L. K. (2012). The analysis of human gastric pacemaker activity. The Journal of Physiology, 590(5), 1299–1300. 10.1113/jphysiol.2011.224014 22399822 PMC3381832

[bph15943-bib-0042] Ördög, T. (2008). Interstitial cells of Cajal in diabetic gastroenteropathy. Neurogastroenterology and Motility, 20, 8–18. 10.1111/j.1365-2982.2007.01056.x 18173559

[bph15943-bib-0043] Ouyang, H. , Xing, J. , & Chen, J. D. Z. (2005). Tachygastria induced by gastric electrical stimulation is mediated via α‐and β‐adrenergic pathway and inhibits antral motility in dogs. Neurogastroenterology and Motility, 17, 846–853. 10.1111/j.1365-2982.2005.00696.x 16336500

[bph15943-bib-0044] Owaki, H. , Sadahiro, S. , & Takaki, M. (2015). Characterizations of the α_1_‐adrenoceptor subtypes mediating contractions of the human internal anal sphincter. Journal of Pharmacological Sciences, 127, 424–429. 10.1016/j.jphs.2015.02.016 25913761

[bph15943-bib-0045] Pal, A. , Indireshkumar, K. , Schwizer, W. , Abrahamsson, B. , Fried, M. , & Brasseur, J. G. (2004). Gastric flow and mixing studied using computer simulation. Proceedings of the Royal Society of London. Series B: Biological Sciences, 271, 2587–2594. 10.1098/rspb.2004.2886 PMC1691895 15615685

[bph15943-bib-0046] Parkman, H. P. , Hallinan, E. K. , Hasler, W. L. , Farrugia, G. , Koch, K. L. , Calles, J. , Snape, W. J. , Abell, T. L. , Sarosiek, I. , McCallum, R. W. , Nguyen, L. , Pasricha, P. J. , Clarke, J. , Miriel, L. , Lee, L. , Tonascia, J. , Hamilton, F. , & NIDDK Gastroparesis Clinical Research Consortium (GpCRC) . (2016). Nausea and vomiting in gastroparesis: Similarities and differences in idiopathic and diabetic gastroparesis. Neurogastroenterology and Motility, 28, 1902–1914. 27350152 10.1111/nmo.12893 PMC5125878

[bph15943-bib-0047] Price, C. J. , Hoyda, T. D. , & Ferguson, A. V. (2008). The area postrema: A brain monitor and integrator of systemic autonomic state. The Neuroscientist, 14, 182–194. 10.1177/1073858407311100 18079557

[bph15943-bib-0048] Rhee, P.‐L. , Lee, J. Y. , Son, H. J. , Kim, J. J. , Rhee, J. C. , Kim, S. , Koh, S. D. , Hwang, S. J. , Sanders, K. M. , & Ward, S. M. (2011). Analysis of pacemaker activity in the human stomach. The Journal of Physiology, 589(24), 6105–6118. 22005683 10.1113/jphysiol.2011.217497 PMC3286689

[bph15943-bib-0049] Rowe, J. W. , Shelton, R. L. , Helderman, J. H. , Vestal, R. , & Robertson, G. L. (1979). Influence of the emetic reflex on vasopressin release in man. Kidney International, 16, 729–735. 10.1038/ki.1979.189 548611

[bph15943-bib-0050] Sanders, K. M. , Ward, S. M. , & Koh, S. D. (2014). Interstitial cells: Regulators of smooth muscle function. Physiological Reviews, 94, 859–907. 10.1152/physrev.00037.2013 24987007 PMC4152167

[bph15943-bib-0051] Sanger, G. J. , & Andrews, P. L. R. (2018). A history of drug discovery for treatment of nausea and vomiting and the implications for future research. Frontiers in Pharmacology, 9, 913. 10.3389/fphar.2018.00913 30233361 PMC6131675

[bph15943-bib-0052] Sanger, G. J. , Broad, J. , Kung, V. , & Knowles, C. H. (2013). Translational neuropharmacology: The use of human isolated gastrointestinal tissues. British Journal of Pharmacology, 168, 28–43. 10.1111/j.1476-5381.2012.02198.x 22946540 PMC3570000

[bph15943-bib-0053] Serradeil‐Le Gal, C. , Wagnon, J. , Garcia, C. , Lacour, C. , Guiraudou, P. , Christophe, B. , Villanova, G. , Nisato, D. , Maffrand, J. P. , & Le Fur, G. (1993). Biochemical and pharmacological properties of SR 49059, a new, potent, nonpeptide antagonist of rat and human vasopressin V1a receptors. The Journal of Clinical Investigation, 92, 224–231. 10.1172/JCI116554 8392086 PMC293574

[bph15943-bib-0054] Sinn, B.‐H. , Sinn, D. H. , Ko, E.‐J. , Lee, J. Y. , Kim, J. J. , Rhee, J. C. , Kim, S. , Ward, S. M. , & Rhee, P.‐L. (2010). Regional differences of the effects of acetylcholine in the human gastric circular muscle. The American Journal of Physiology, 299, G1198–G1203. 10.1152/ajpgi.00523.2009 20798355

[bph15943-bib-0055] Smit, T. , Kotze, I. , & du Plessis, J. (2021). The incidence of nausea in the absence of vomiting in patients receiving intravenous chemotherapy. Ann Palliat Med, 10, 2679–2686. 10.21037/apm-19-453 33549001

[bph15943-bib-0056] Stern, R. M. , Koch, K. L. , & Andrews, P. L. R. (2011). Nausea: Mechanisms and management (Vol. 2011). Oxford University Press.

[bph15943-bib-0057] Straface, M. , Koussai, M.‐A. , Makwana, R. , Crawley, E. , Palmer, A. , Cai, W. , Gharibans, A. , Adebibe, M. , Loy, J. , O'Grady, G. , Andrews, P. L. R. , & Sanger, G. J. (2022). A multi‐parameter approach to measurement of spontaneous myogenic contractions in human stomach: Utilization to assess potential modulators of myogenic contractions. Pharmacological Research, 180, 106247. 10.1016/j.phrs.2022.106247 35533804

[bph15943-bib-0058] Verbalis, J. G. , Richardson, D. W. , & Stricker, E. M. (1987). Vasopressin release in response to nausea‐producing agents and cholecystokinin in monkeys. The American Journal of Physiology, 252, R749–R753. 3032008 10.1152/ajpregu.1987.252.4.R749

[bph15943-bib-0059] Xu, L. H. , Koch, K. L. , Summy‐Long, J. , Stern, R. M. , Seaton, J. F. , Harrison, T. S. , Demers, L. M. , & Bingaman, S. (1993). Hypothalamic and gastric myoelectrical responses during vection‐induced nausea in healthy Chinese subjects. The American Journal of Physiology, 265, E578–E584. 8238333 10.1152/ajpendo.1993.265.4.E578

[bph15943-bib-0060] Yin, J. , & Chen, J. D. Z. (2013). Electrogastrography: Methodology, validation and applications. Neurogastroenterology and Motility, 19, 5–17. 10.5056/jnm.2013.19.1.5 PMC3548127 23350042

