# Supplementary material for: Synergistic augmentation of rhythmic myogenic contractions of human stomach by arginine vasopressin and adrenaline: Implications for the induction of nausea
Source: Br J Pharmacol. 2022 Sep 22;179(24):5305–22. doi: 10.1111/bph.15943 (PMC9826163; doi:10.1111/bph.15943)
Supplement: Supplementary file 1 — Figure S1: Comparison between Glyceraldehyde 3‐phosphate dehydrogenase (GAPDH) mRNA levels in human proximal and distal stomach muscle. (B) Expression of AVP, OT and ADr receptor mRNA in human proximal (P) and distal (D) stomach muscle normalised to GAPDH mRNA levels. Ct is inversely proportional to level of gene expression. Data are mean ± S.E.M.; n = 5–8. Figure S2: Comparison between the various features of the spontaneous contraction waveform of male (n = 7) and female (n = 37) human (A) proximal and (B) distal stomach circular muscle, and of (C) proximal and (D) distal stomach circular muscle from people with (n = 9) and without (n = 32) diabetes (n = 2 patient records unavailable). Also shown are the maximal increases in muscle tone caused by carbachol (10−3 M). Each value represents the mean ± S.E.M. Total n = 44 donors. Figure S3: Comparison between the various features of the spontaneous contraction waveform and baseline muscle tone of human (A) proximal and (B) distal stomach circular muscle before (Baseline) and after 30 min incubation with atropine (10−6 M). Each value represents the mean ± S.E.M. n = 6. Figure S4: Spontaneous contractions of human (A) proximal and (B) distal stomach circular muscle in the absence and presence of a single challenge of individual strips with AVP at the concentration indicated. (C) Comparison of the AVP‐induced increase in muscle tone (I,ii), and the amplitude (iii,iv) and frequency (v,vii) of spontaneous contractions of human proximal (graphs on the left) and distal (graphs on the right) stomach by single or cumulative application of AVP. (D) Time course profile for the AVP‐induced increase in muscle tone (I,ii), and the amplitude (iii,iv) and frequency (v,vii) of spontaneous contractions of human proximal (graphs on the left) and distal (graphs on the right) stomach. Data are mean ± S.E.M.; n = 4. Figure S5: (A) AVP‐ and (B) OT‐induced increase in muscle tone (i, ii) and the amplitude (iii, iv) and frequency (v, vi) of s [file BPH-179-5305-s003.pdf]

`A

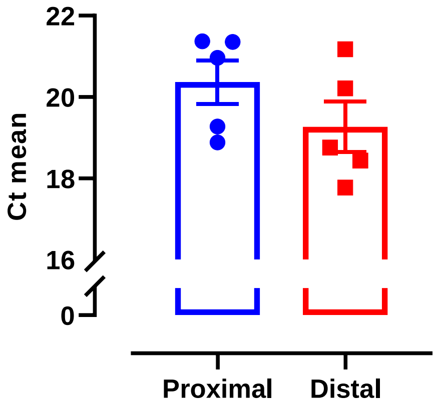

B

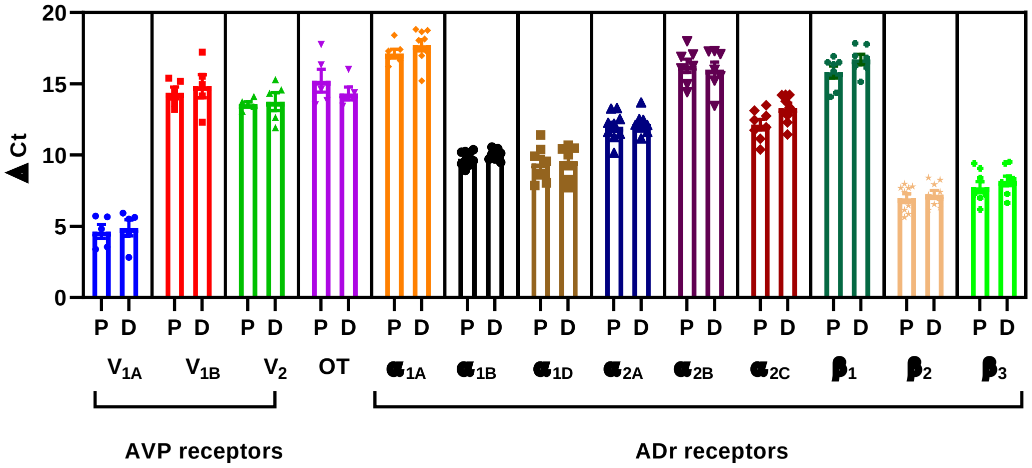

Figure S1: Comparison between Glyceraldehyde 3-phosphate dehydrogenase (GAPDH) mRNA levels in human proximal and distal stomach muscle. (B) Expression of AVP, OT and ADr receptor mRNA in human proximal (P) and distal (D) stomach muscle normalised to GAPDH mRNA levels. ΔCt is inversely proportional to level of gene expression. Data are mean±S.E.M. n=5-8.

A

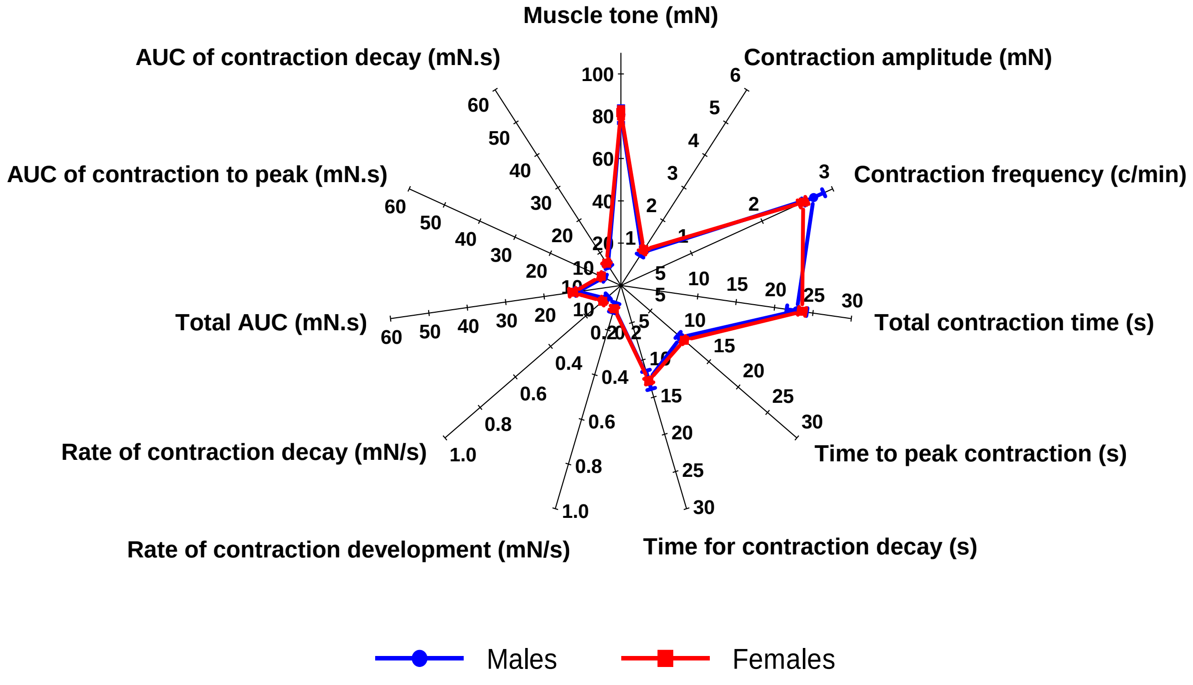
Muscle tone (mN)
Contraction amplitude (mN)
Contraction frequency (c/min)
Total contraction time (s)
Time to peak contraction (s)
Time for contraction decay (s)
Rate of contraction development (mN/s)
Rate of contraction decay (mN/s)
Total AUC (mN.s)
AUC of contraction to peak (mN.s)
AUC of contraction decay (mN.s)
Males
Females

B

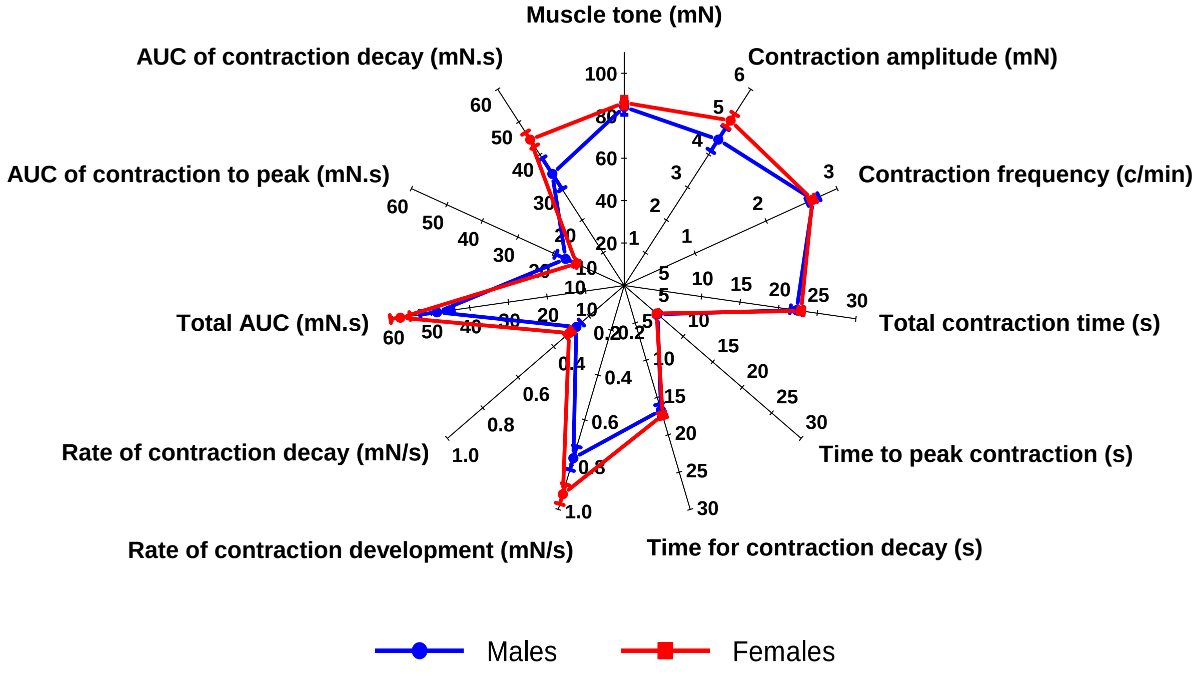
Muscle tone (mN)
Contraction amplitude (mN)
Contraction frequency (c/min)
Total contraction time (s)
Time to peak contraction (s)
Time for contraction decay (s)
Rate of contraction development (mN/s)
Rate of contraction decay (mN/s)
Total AUC (mN.s)
AUC of contraction to peak (mN.s)
AUC of contraction decay (mN.s)
Males
Females

C

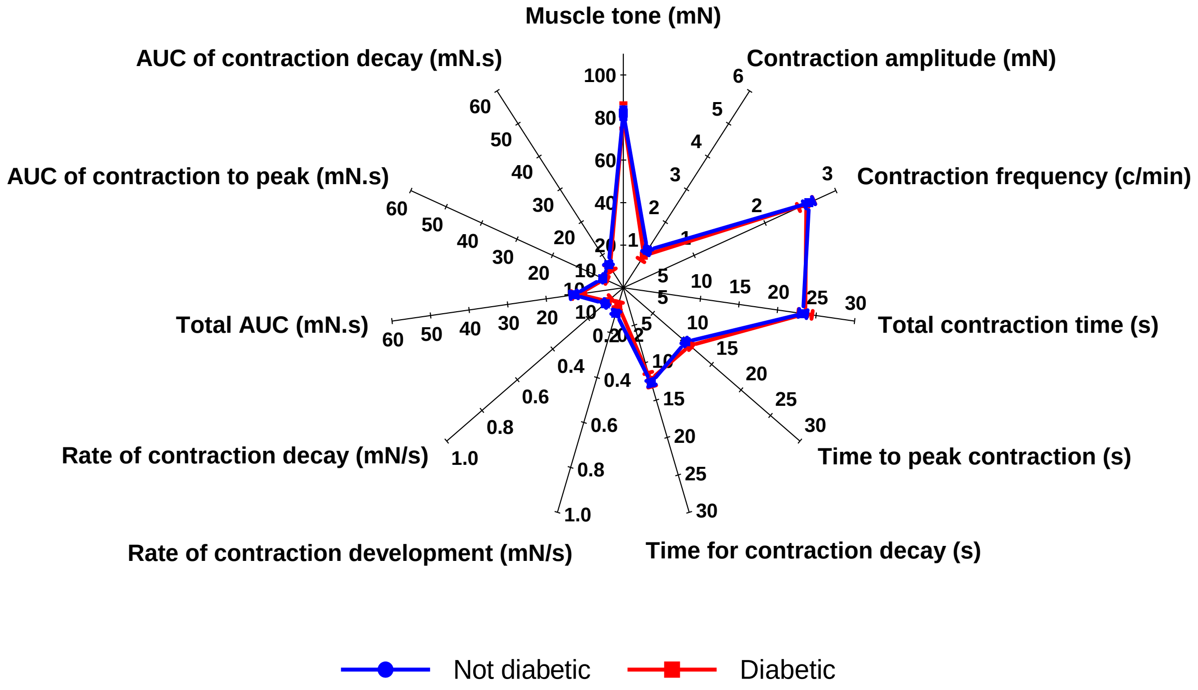

D

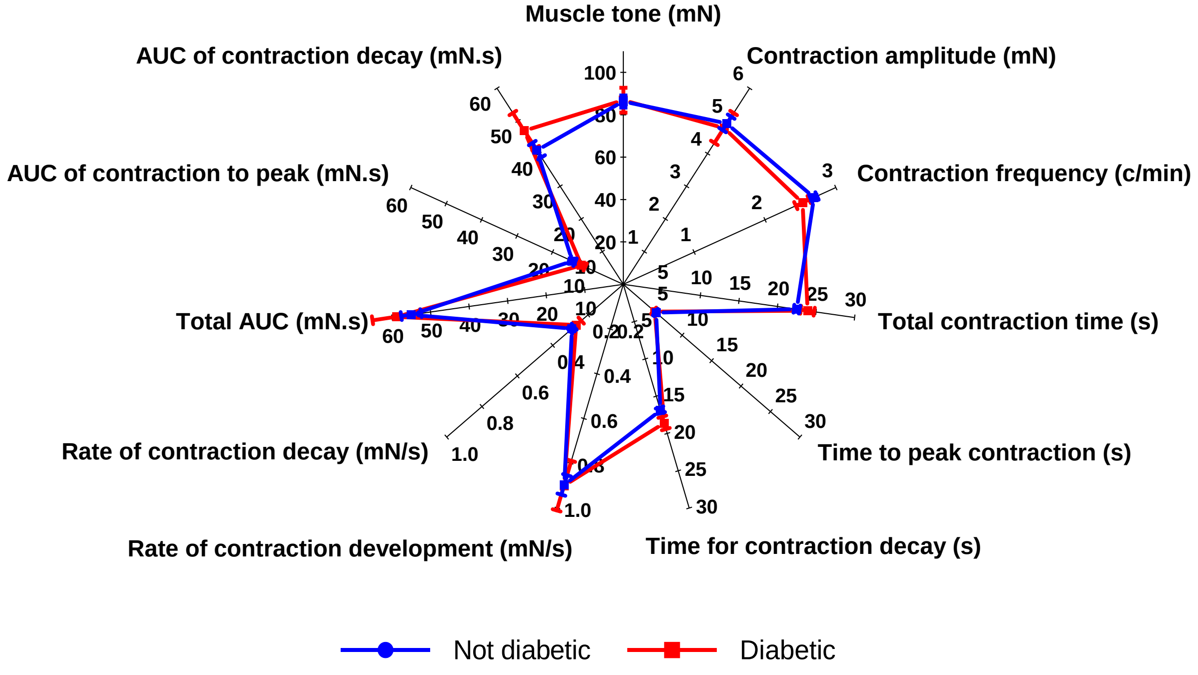

Figure S2: Comparison between the various features of the spontaneous contraction waveform of male (n=7) and female (n=37) human (A) proximal and (B) distal stomach circular muscle, and of (C) proximal and (D) distal stomach circular muscle from people with (n=9) and without (n=32) diabetes (n=2 patient records unavailable). Also shown are the maximal increases in muscle tone caused by carbachol (10^{-3}M). Each value represents the mean±S.E.M. Total n=44 donors.

A

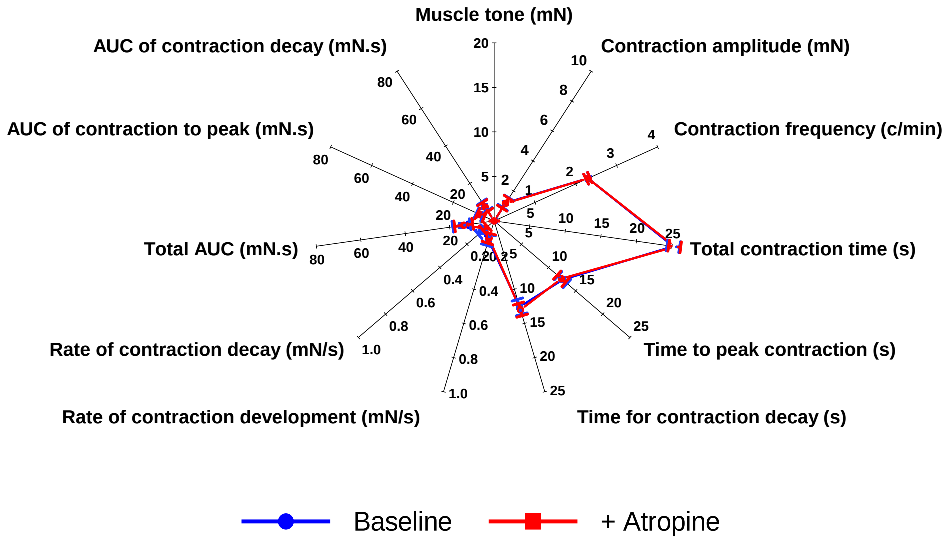

B

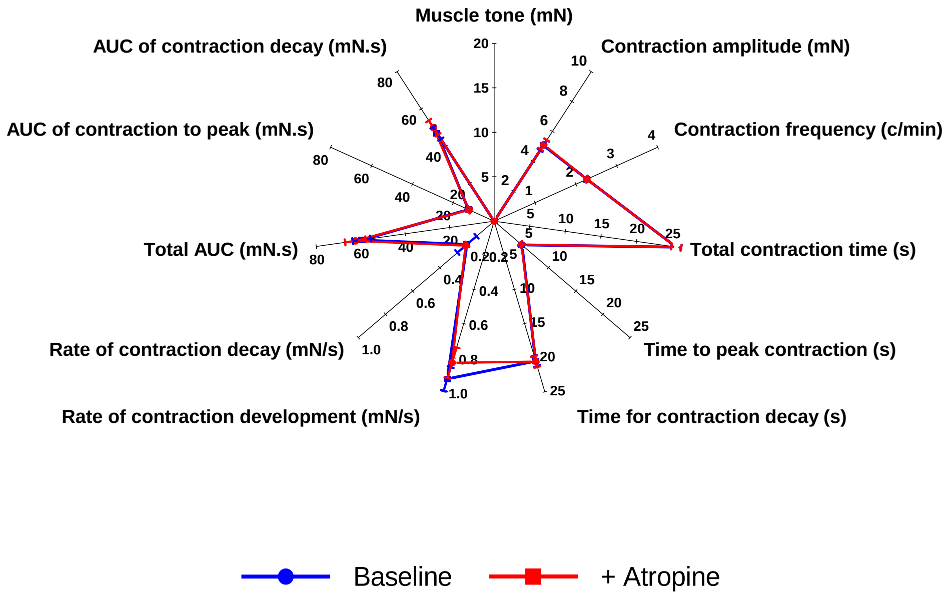

Figure S3: Comparison between the various features of the spontaneous contraction waveform and baseline muscle tone of human (A) proximal and (B) distal stomach circular muscle before (Baseline) and after 30 min incubation with atropine (10^{-6}M). Each value represents the mean±S.E.M. n=6.

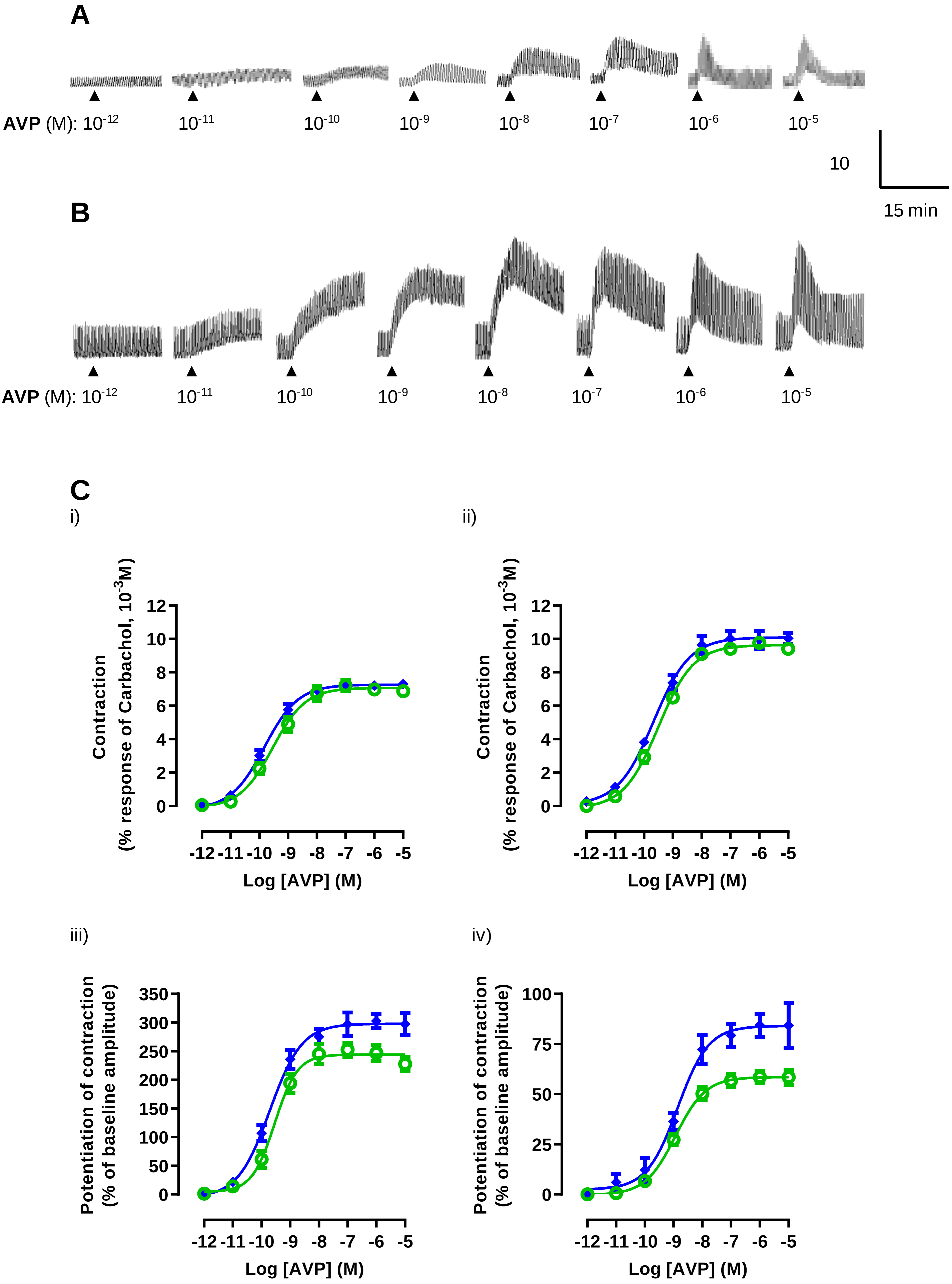

A
AVP (M): 10-12
10-11
10-10
10-9
10-8
10-7
10-6
10-5
10
15 min
B
AVP (M): 10-12
10-11
10-10
10-9
10-8
10-7
10-6
10-5
C
i)
ii)
Contraction
(% response of Carbachol, 10-3M)
Log [AVP] (M)
iii)
iv)
Potentiation of contraction
(% of baseline amplitude)
Log [AVP] (M)

v) vi)

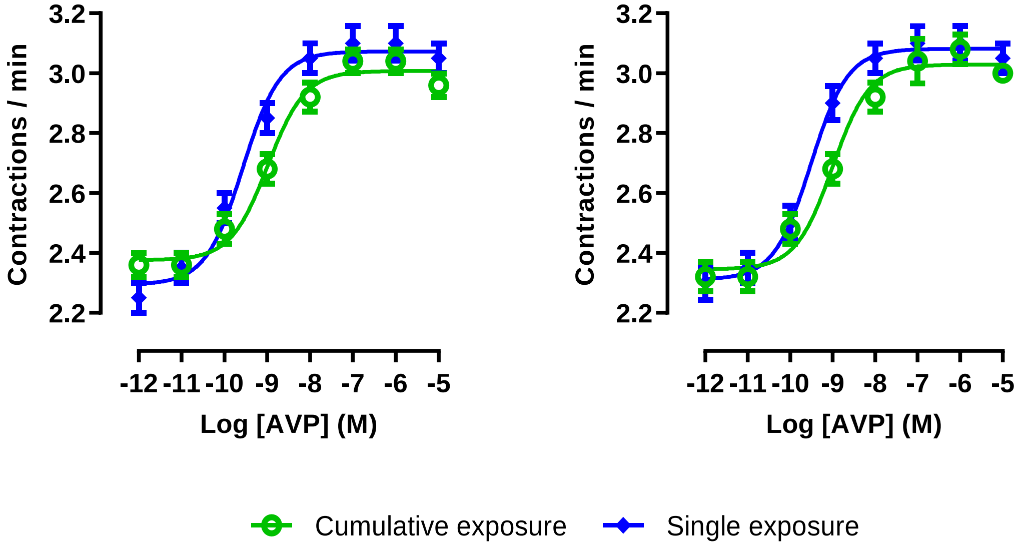

D

i)

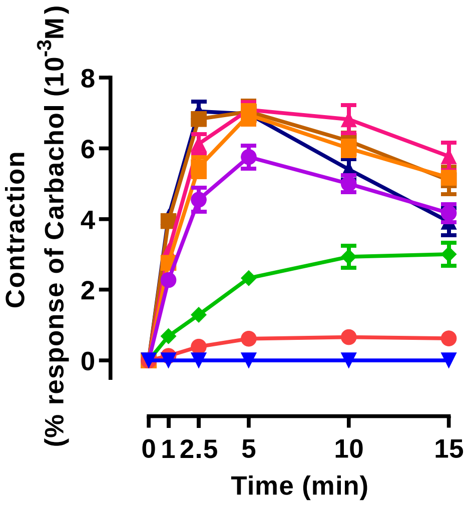

ii)

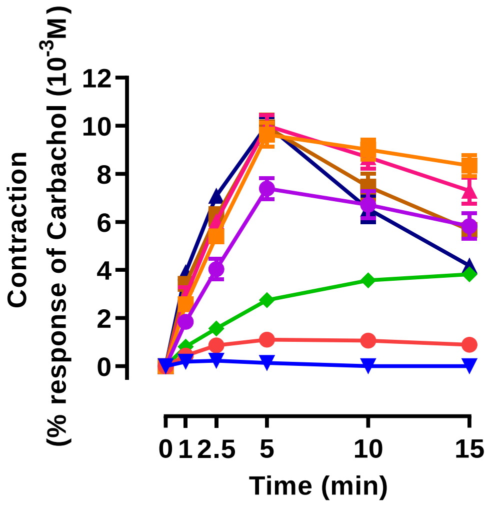

iii)

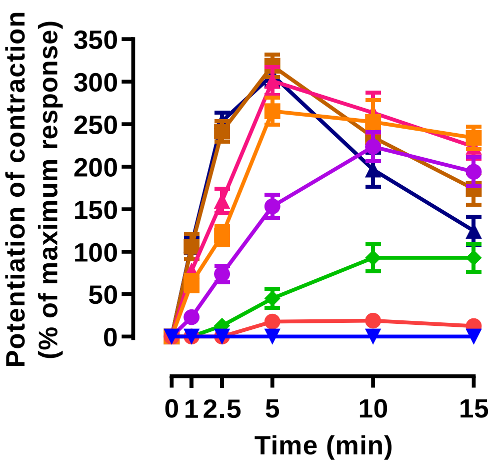

iv)

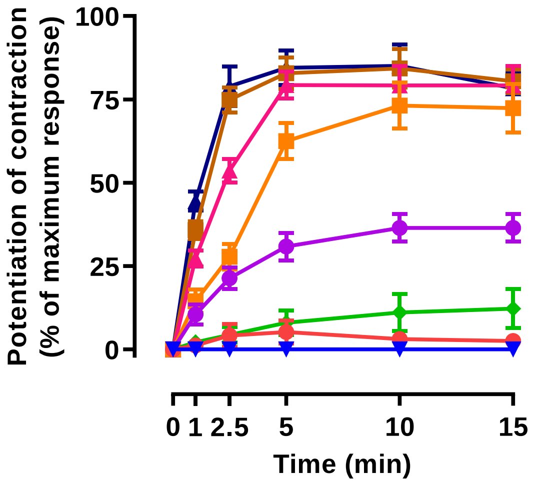

v) vi)

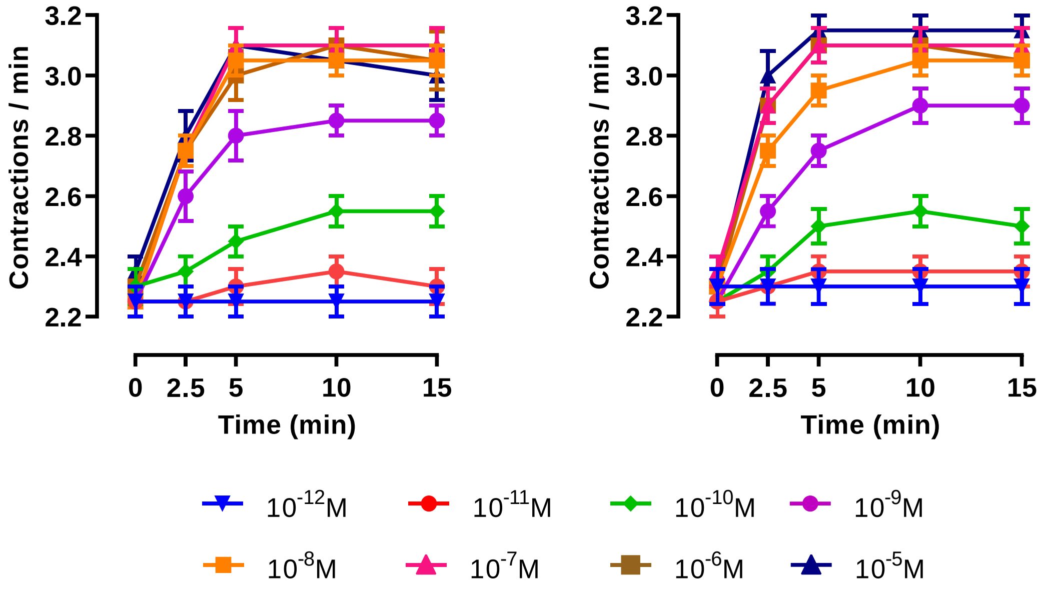

Figure S4: Spontaneous contractions of human (A) proximal and (B) distal stomach circular muscle in the absence and presence of a single challenge of individual strips with AVP at the concentration indicated. (C) Comparison of the AVP-induced increase in muscle tone (I,ii), and the amplitude (iii,iv) and frequency (v,vii) of spontaneous contractions of human proximal (graphs on the left) and distal (graphs on the right) stomach by single or cumulative application of AVP. (D) Time course profile for the AVP-induced increase in muscle tone (I,ii), and the amplitude (iii,iv) and frequency (v,vii) of spontaneous contractions of human proximal (graphs on the left) and distal (graphs on the right) stomach. Data are mean±S.E.M. n=4.

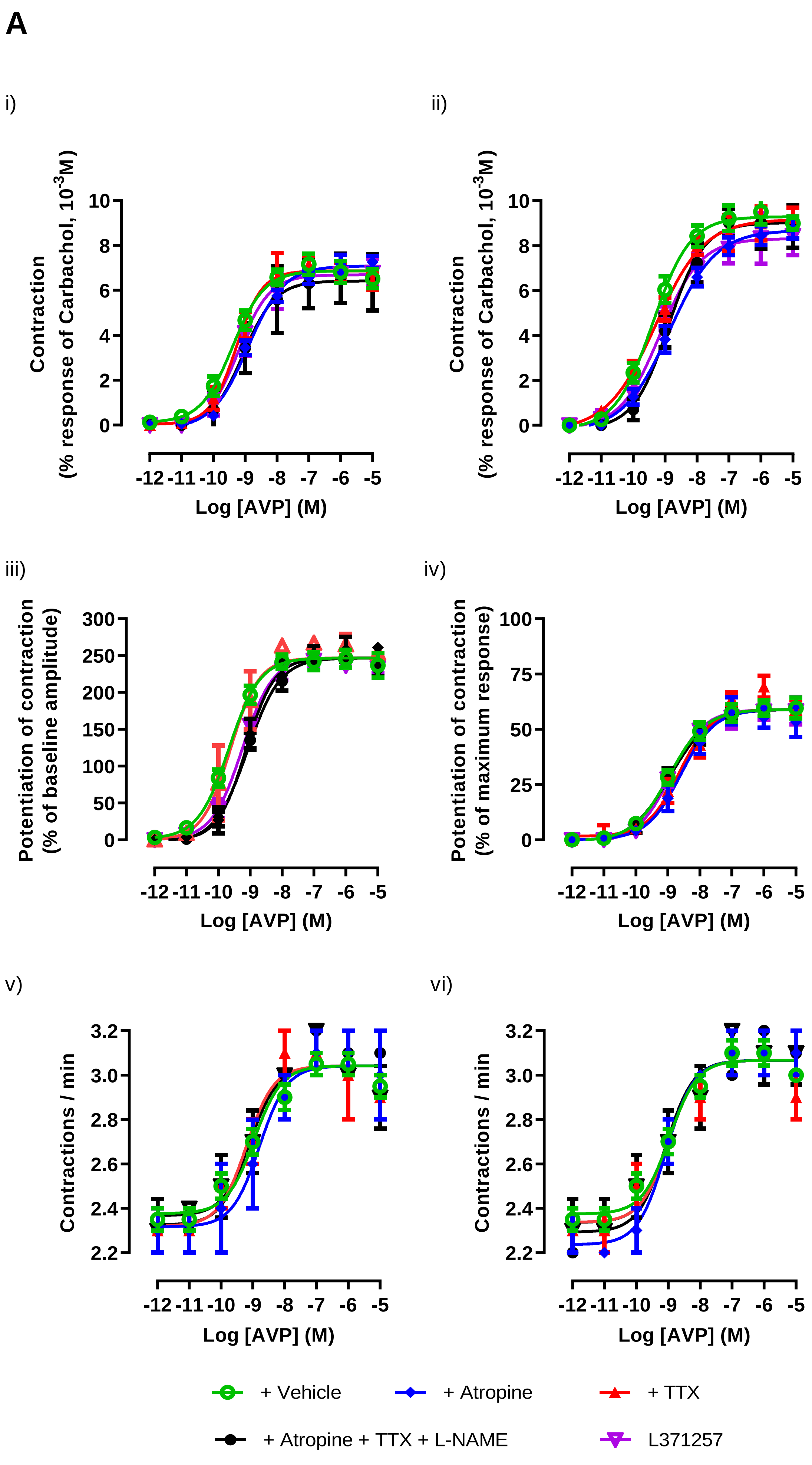
A
i)
ii)
iii)
iv)
v)
vi)
Contraction (% response of Carbachol, 10-3M)
Potentiation of contraction (% of baseline amplitude)
Potentiation of contraction (% of maximum response)
Contractions / min
Log [AVP] (M)
+ Vehicle
+ Atropine
+ TTX
+ Atropine + TTX + L-NAME
L371257

B

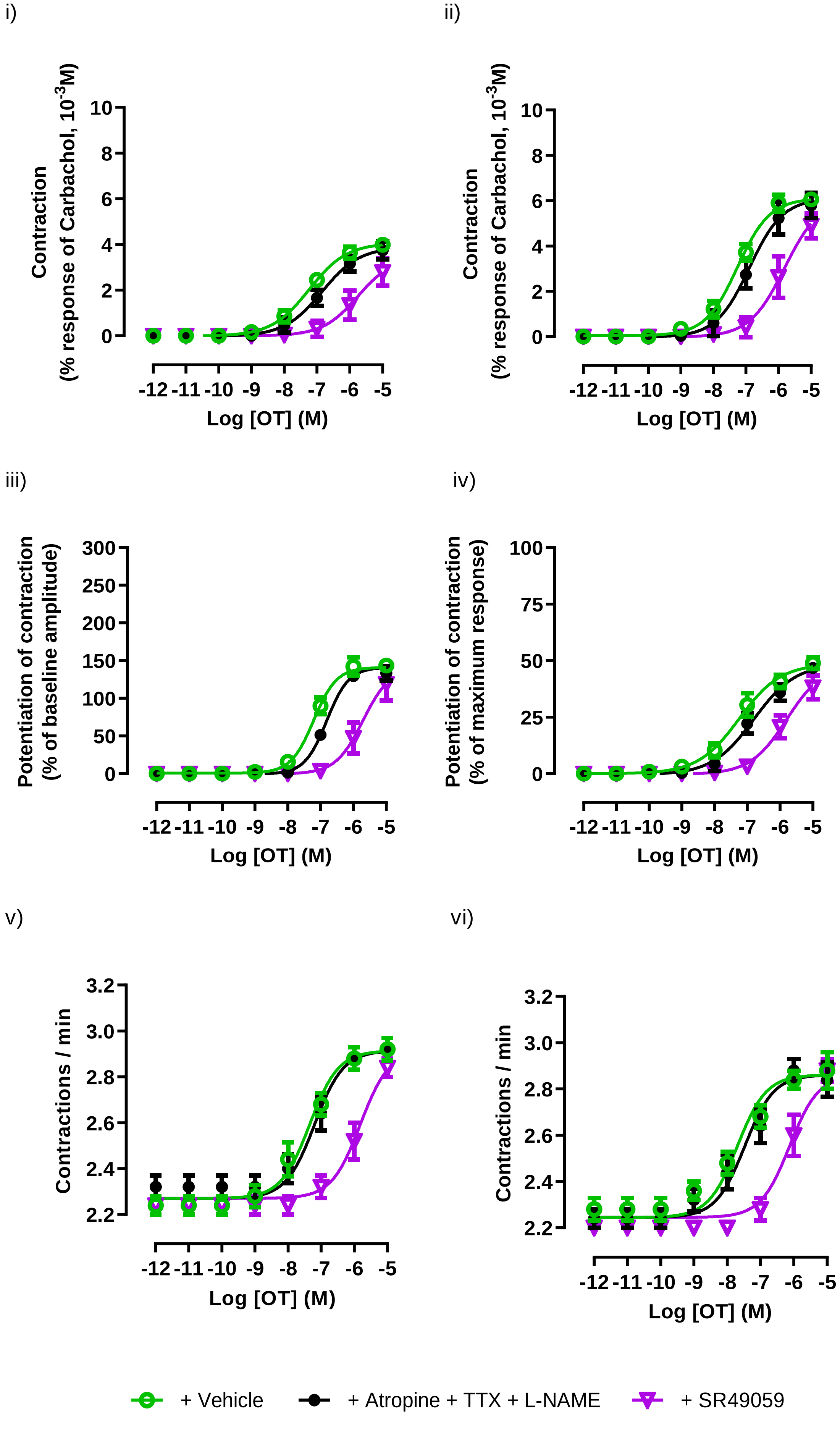

i)
ii)
iii)
iv)
v)
vi)
Contraction
(% response of Carbachol, 10^-3M)
Potentiation of contraction
(% of baseline amplitude)
Potentiation of contraction
(% of maximum response)
Contractions / min
Log [OT] (M)
-12 -11 -10 -9 -8 -7 -6 -5
+ Vehicle
+ Atropine + TTX + L-NAME
+ SR49059

Figure S5: (A) AVP- and (B) OT-induced increase in muscle tone (i, ii) and the amplitude (iii, iv) and frequency (v, vi) of spontaneous contractions of human proximal (graphs on the left) and distal (graphs on the right) stomach, after a 30 min incubation with either TTX (10^{-6}M), atropine (10^{-6}M), a combination of tetrodotoxin (10^{-6}M), atropine (10^{-6}M) and L-NAME (3 x10^{-4}M), SR49059 (10^{-8}M) or L371257(10^{-7}M) or their vehicle (DMSO and water, 0.5%v/v). Data are mean±S.E.M, n=5.

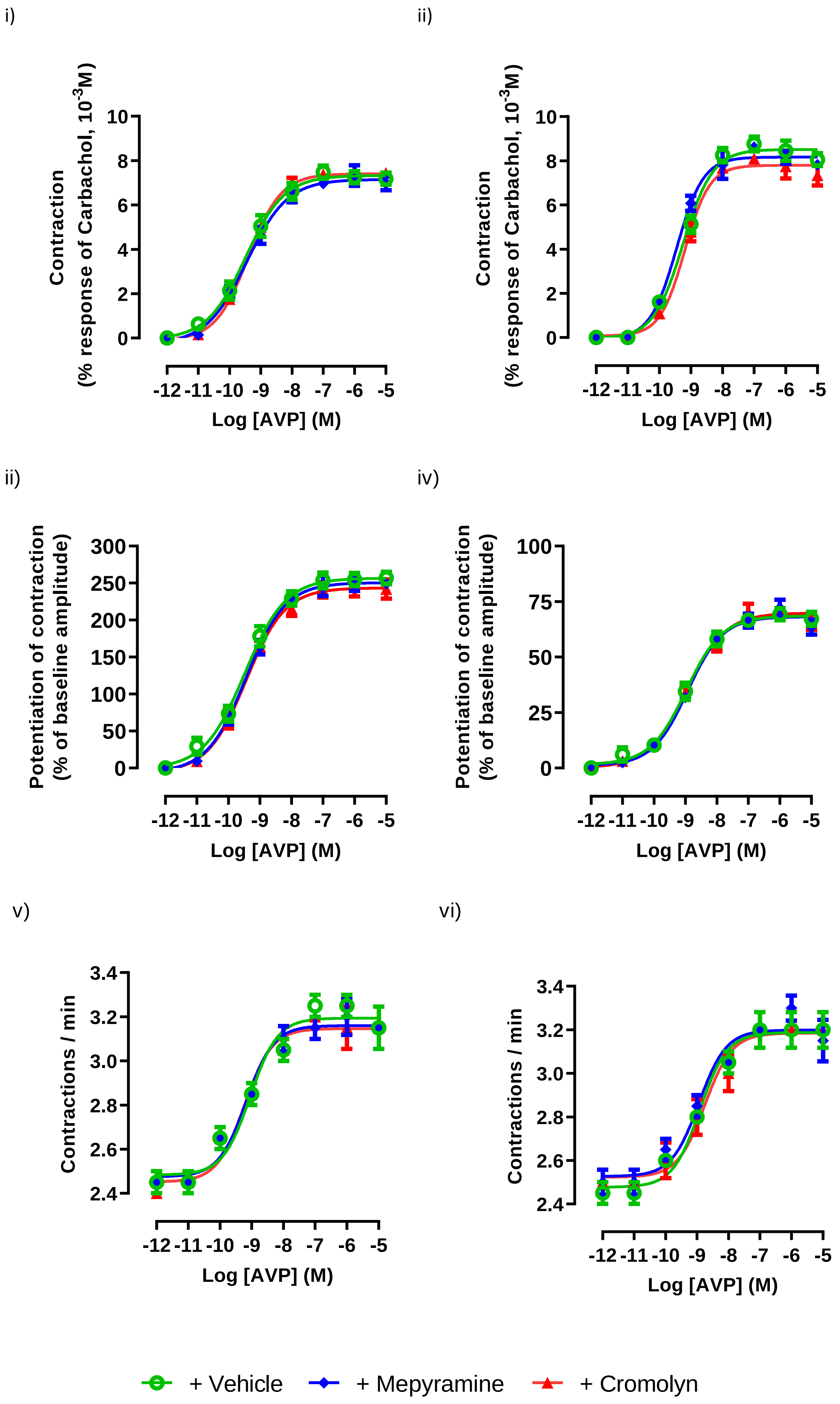

i)
Contraction
(% response of Carbachol, 10-3M)
0
2
4
6
8
10
-12 -11 -10 -9 -8 -7 -6 -5
Log [AVP] (M)
ii)
Contraction
(% response of Carbachol, 10-3M)
0
2
4
6
8
10
-12 -11 -10 -9 -8 -7 -6 -5
Log [AVP] (M)
ii)
Potentiation of contraction
(% of baseline amplitude)
0
50
100
150
200
250
300
-12 -11 -10 -9 -8 -7 -6 -5
Log [AVP] (M)
iv)
Potentiation of contraction
(% of baseline amplitude)
0
25
50
75
100
-12 -11 -10 -9 -8 -7 -6 -5
Log [AVP] (M)
v)
Contractions / min
2.4
2.6
2.8
3.0
3.2
3.4
-12 -11 -10 -9 -8 -7 -6 -5
Log [AVP] (M)
vi)
Contractions / min
2.4
2.6
2.8
3.0
3.2
3.4
-12 -11 -10 -9 -8 -7 -6 -5
Log [AVP] (M)
+ Vehicle
+ Mepyramine
+ Cromolyn

Figure S6: AVP-induced increase in muscle tone (i, ii) and amplitude (iii, iv) and frequency (v, vi) of spontaneous contractions of human proximal (graphs on left) and distal (right) stomach, after 30min incubation with mepyramine (10^{-7}M), cromolyn (10^{-5}M) or their vehicle (water 0.1%v/v). Data are mean±S.E.M. n=4.

A)

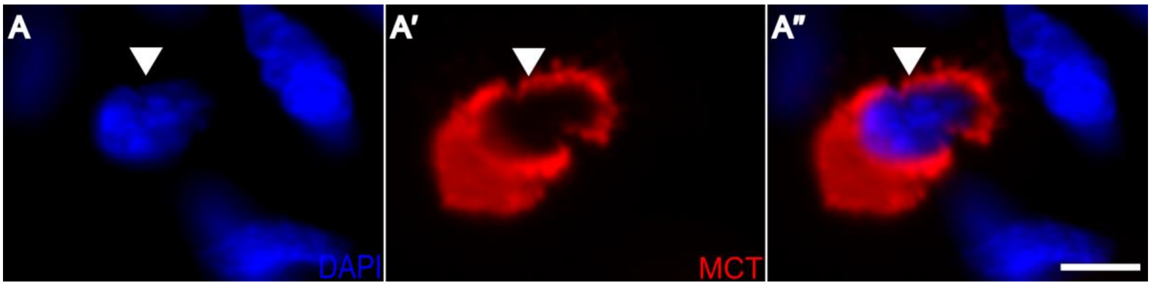

B)

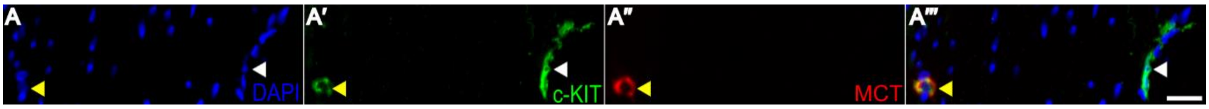

Figure S7: (A) Representative mast cell staining in distal stomach muscle. Image A: Mast cell was counterstained for 4′,6-diamidino-2-phenylindole (DAPI) (white arrow). Image A' and A'' show the cytoplasmic localisation of the mast cell tryptase (MCT) stain (A'' includes DAPI stain). Images were captured at 40x magnification The scale bar 5 µm. (B) Distinguishing between mast cells and interstitial cells of Cajal (ICC) in distal stomach muscle. A: DAPI counterstained cells. Images A' and A'' highlight a mast cell (yellow arrow) staining for respectively, c-KIT, and mast cell tryptase (MCT). Image A''' shows the merged image together with an ICC (white arrow) staining for c-KIT; extensions, when present, were only seen protruding from the ICC whereas mast cells often appeared with 'rounder' nuclei. Images were captured at 20x magnification The white scale bar is 10 µm. All images were taken using an Olympus BX61 microscope and SmartCapture3 software. MCT antibody used for preliminary studies was Agilent Dako (M705229-2), c-KIT antibody used was Agilent Dako (A4502). ICC and Mast cell differentiation criteria was confirmed for multiple cells in separate images from 4 different donors using the same primary antibodies whilst optimising other staining conditions.

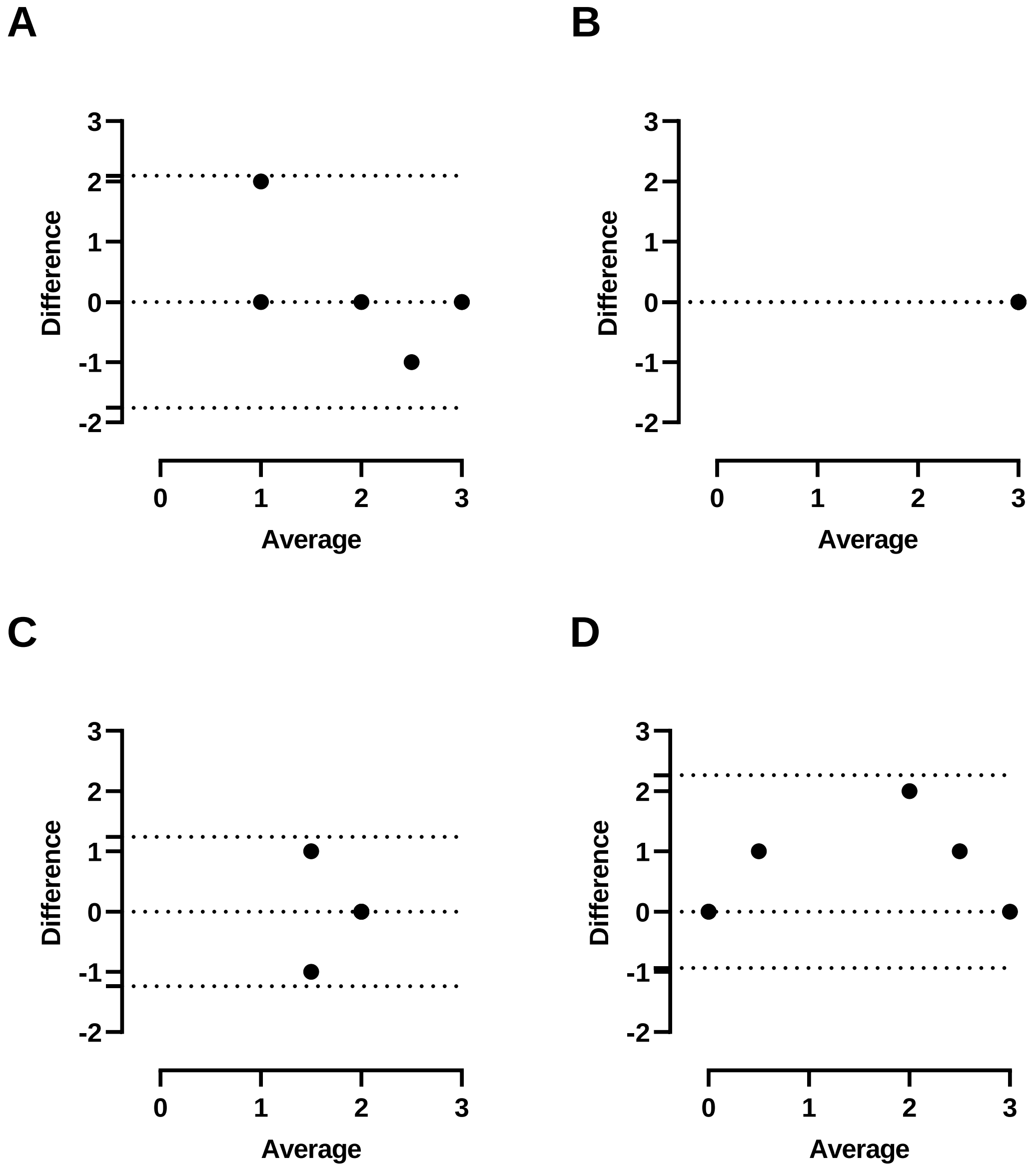

Figure S8: Bland-Altman (average vs difference) analyses showing lack of inter-rater bias between two investigators observing immunofluorescence for the V_{1A} receptor on (A) c-Kit and (B) α-SMA -positive cells and α_1 adrenoceptor on (C) c-Kit and (D) α-SMA -positive cells. c-Kit and α-SMA positive cells indicated ICCs and myocytes, respectively. Analyses was performed on immunofluorescence images of three randomly selected cells of both cell types in the distal stomach muscle layer of 6 people. Dotted lines above and below origin (0) are 95% confidence intervals.
