# Supplementary material for: Synergistic augmentation of rhythmic myogenic contractions of human stomach by arginine vasopressin and adrenaline: Implications for the induction of nausea
Source: Br J Pharmacol. 2022 Sep 22;179(24):5305–22. doi: 10.1111/bph.15943 (PMC9826163; doi:10.1111/bph.15943)
Supplement: Supplementary file 2 — Data S1 Supporting information [file BPH-179-5305-s002.docx]

**Supplementary Methods**

1. **Expression of V_1A_, V_1B_, V_2_, OT and α_1A,B,D_, α_2A-C_, β_1-3_ receptor mRNA in human stomach investigated using qPCR**

mRNA expression was investigated using qPCR. Briefly, 0.5cm^2^ proximal and distal stomach muscle (4 male, 4 female; 2/8 were diabetic; 28-54, median 41 years, stored at -80ºC in RNA*later*^TM (^Sigma-Aldrich, Gillingham, UK) was homogenized in TRI Reagent^®^ ^(^Sigma-Aldrich, Gillingham, UK) and total RNA extracted (Direct-zol RNA Miniprep kit; Zymo Research. California, USA). cDNA was reverse transcribed from RNA (10^-7^g) (SuperScript^TM^ VILO^TM^ cDNA Synthesis kit; ThermoFisher, Kent, UK) and the concentration of each receptor gene amplified by 40 cycles, quantified (SYBR^®^ Green reagents; ThermoFisher, Kent, UK) on a StepOne^TM^ real-time PCR system (Applied Biosystems, California, USA) and analyzed (The Expression Suite v1.2 software; ThermoFisher, Kent, UK.). Gene expression was normalized against glyceraldehyde 3-phosphate dehydrogenase (GAPDH) and quantified by the change in cycle threshold (ΔCt) method. Oligonucleotides (primers) were used at 4x10^-5^M and Table SM3 lists the primer sequences and their suppliers.

qPCR oligonucleotide (primer) characteristics

| **Gene** | **Specifics** |
| --- | --- |
| **GAPDH** | Forward Primer Sequence (5' - 3'): CTCTGCTCCTCCTGTTCGAC  Reverse Primer Sequence (5' - 3'): TTAAAAGCAGCCCTGGTGAC  Region Amplified (bp) 614 – 717  Exon-Exon Boundary 1 – 3  Amplicon Size (bp) 144/236  Supplier: Sigma-Aldrich, Gillingham, UK |
| **AVPR1A** | Forward Primer Sequence (5' - 3'): GATCGCTGATTCATTGGACAACA  Reverse Primer Sequence (5' - 3'): TGTGGGTGGCTTTGATCTACC  Accession Number: NM_000706.4  Anchor nucleotide: 4681  Amplicon Size (bp): 128  Supplier: Primerdesign Ltd, Chandler’s Ford, UK |
| **AVPR1B** | Forward Primer Sequence (5' - 3'): TGTCCACTTTTGTCCTCATCTGG  Reverse Primer Sequence (5' - 3'): GTGCCATGCTCTTCCTCTACC  Accession Number: NM_000707.4  Anchor Nucleotide: 2743  Amplicon Size (bp): 97  Supplier: Primerdesign Ltd, Chandler’s Ford, UK |
| **AVPR2** | Forward Primer Sequence (5' - 3'): GAGGAGTGGCAGGAAAGAGG  Reverse Primer Sequence (5' - 3'): GAATGAGGAGGGAGGGATTAGAA  Accession Number: NM_000054  Anchor Nucleotide: 1666  Amplicon Size (bp): 131  Supplier: Primerdesign Ltd, Chandler’s Ford, UK |
| **OXTR** | Forward Primer Sequence (5' - 3'): TTACAATCACTAGGATGGCTACAA  Reverse Primer Sequence (5' - 3'): CATTTACATTCCCACCAACAATTTAA  Accession Number: NM_000916  Anchor Nucleotide: 3744  Amplicon Size (bp): 105  Supplier: Primerdesign Ltd, Chandler’s Ford, UK |
| **ADRA1A** | Forward Primer Sequence (5' - 3'): CCTTCTACAGGATCTCCAAG  MW: 6037, T_m_: 57.9^O^C  Reverse Primer Sequence (5' - 3'): TATCTACAATCCATTCCCCTC  MW: 6236, T_m_: 59.3^O^C  Supplier: Sigma-Aldrich, Gillingham, UK |
| **ADRA1B** | Forward Primer Sequence (5' - 3'): ATATAGTGGCCAAGAGAACC  MW: 6159, T_m_: 57.2^O^C  Reverse Primer Sequence (5' - 3'): TCGTGAAAGTTCTTGGAATG  MW: 6187, T_m_: 60.3^O^C  Supplier: Sigma-Aldrich, Gillingham, UK |
| **ADRA1D** | Forward Primer Sequence (5' - 3'): ACAAGCCTGCTGTATTIATC  MW: 6067, T_m_: 56.0^O^C  Reverse Primer Sequence (5' - 3'): TTGAGGGGAAGTAATAAGGG  MW: 6310, T_m_: 59.5^O^C  Supplier: Sigma-Aldrich, Gillingham, UK |
| **ADRA2A** | Forward Primer Sequence (5' - 3'): ACATGTTGCTAATGACAGTG  MW: 6156, T_m_: 56.0^O^C  Reverse Primer Sequence (5' - 3'): AGATAACAGACAAGAGGACC  MW: 6177, T_m_: 54.7^O^C  Supplier: Sigma-Aldrich, Gillingham, UK |
| **ADRA2B** | Forward Primer Sequence (5' - 3'): GAGACCCCTGAAGATACTG  MW: 5822, T_m_: 56.1^O^C  Reverse Primer Sequence (5' - 3'): CTCCTCTTCCTCCTCTTC  MW: 5263, T_m_: 55.3^O^C  Supplier: Sigma-Aldrich, Gillingham, UK |
| **ADRA2C** | Forward Primer Sequence (5' - 3'): AGTTCTTCTTCTGGATCGG  MW: 5801, T_m_: 58.4^O^C  Reverse Primer Sequence (5' - 3'): GAAGAGGATGTGCTTAAAGG  MW: 6270, T_m_: 58.0^O^C  Supplier: Sigma-Aldrich, Gillingham, UK |
| **ADRAB1** | Forward Primer Sequence (5' - 3'): AAAAGGAAAGTTTGGGAAGG  MW: 6303, T_m_: 60.9^O^C  Reverse Primer Sequence (5' - 3'): CTCAGAGAGTGTCAAAAACC  MW: 6119, T_m_: 56.4^O^C  Supplier: Sigma-Aldrich, Gillingham, UK |
| **ADRAB2** | Forward Primer Sequence (5' - 3'): CACTCCTCTTATTTGCTCAC  MW: 5954, T_m_: 56.5^O^C  Reverse Primer Sequence (5' - 3'): AAACTTTAGACTTTGCTCGG  MW: 6107, T_m_: 57.7^O^C  Supplier: Sigma-Aldrich, Gillingham, UK |
| **ADRAB3** | Forward Primer Sequence (5' - 3'): ATGAGACCTTAGTGTTCTCC  MW: 6083, T_m_: 54.7^O^C  Reverse Primer Sequence (5' - 3'): CATTCCATGGCTAAAGTGAG  MW: 6141, T_m_: 59.7^O^C  Supplier: Sigma-Aldrich, Gillingham, UK |

Each gene was amplified using the following protocol: 20 s at 95^O^C, 40 cycles of 3s at 95^O^C and 30s at 60^O^C, followed by a melt curve from 15s at 95^O^C, 1 min at 60^O^C and 15s at 95^O^C.

1. **Immunohistochemical localization of cell types and receptors and analysis**

To determine the cellular localization of V_1A_ vasopressin and α_1_ adrenaline receptors, 1cm^2^ samples of full-thickness distal stomach (3 male, 3 female; 40-59, median 47 years, 3/6 diabetic) were fixed in 10% neutral buffered formal saline, embedded in paraffin wax, cross-sectioned into 4μm thick slices and mounted on microscope slides.

Certain antibodies worked optimally when the target receptor/ protein antigen epitope was retrieved under *p*H 6 or *p*H 9 buffer conditions.

1. *Antibodies requiring antigen retrieval at pH 6*

Sections were deparaffinised and rehydrated by respectively, heating in an oven at 60^O^C for 30 min and two 3 min sequential washes in 100% xylene, 100% ethanol before finally being washed for 5 min in distilled water. This was followed by heat-treatment in citrate buffer (*p*H 6) for 10 min in a microwave oven to unmask antigen epitopes, permeabilization with 0.1% v/v Triton X-100 in phosphate-buffered saline (PBS) for 30 min at room temperature and blocking with 1% w/v bovine serum albumin (BSA) in PBS for 90 min at room temperature.

Sections were incubated with primary antibodies targeting either c-Kit and V_1A_ receptor or alpha smooth muscle actin (αSMA) and the V_1A_ receptor. These were diluted in 1% w/v BSA in PBS.

After removal of the primary antibodies with three washes with 1% w/v BSA in PBS, each section was incubated with the appropriate fluorophore-tagged secondary antibodies, each diluted in 1% w/v BSA in PBS. This was followed by 1 min incubation with an auto-florescence quencher (TrueVIEW^®^ Auto-fluorescence Quenching Kit) and a 5 min wash in PBS. Finally, sections were incubated with the cell nuclei counterstain 4′,6-diamidino-2-phenylindole (DAPI) for 10 min at room temperature and then washed again with PBS for 5 min. Coverslips were mounted using VECTASHIELD® Vibrance™ Antifade mounting medium for imaging.

1. *Antibodies requiring antigen retrieval at pH 9*

Sections were deparaffinized and rehydrated by respectively, heating in an oven at 60 ^O^C for 30 min and two 10 min sequential washes in 100% Histoclear, two 2 min washes in 100% ethanol, one 2 min wash in 90%, 70%, 50% and 25% ethanol and two 2 min washes in MilliQ water. This was followed by heat-treatment in Target Retrieval Solution (Tris/ethylene diamine tetra-acetate buffer, *p*H 9) (Agilent Dako) for 25 min in a microwave oven to unmask antigen epitopes, permeabilization with three 10 min sequential washes in Tris Buffered saline Triton X-100 (TBST) at room temperature and blocking with 1% w/v BSA in TBST for 90 min at room temperature.

Sections were subsequently incubated with primary antibodies, targeting either c-Kit and the α_1_ receptor or αSMA and the α_1_ adrenoceptor for ~16 h at 4ºC. After removal of primary antibodies with three 10 min washes with TBST, sections were incubated with the appropriate secondary antibodies for 1 h at room temperature followed by another three 10 min washes in TBST. Sections were incubated for 1 min with the TrueVIEW® auto-florescence quencher, washed for 5 min with TBST. Finally, sections were incubated DAPI for 10 min at room temperature before a wash in TBST for 5 min. Coverslips were mounted as above before imaging.

1. *Imaging and analysis*

Images were captured at x20 or x40 magnification with Smart Capture v3 (DeskSoft, Strasshof an der Nordbahn, Austria) and an Olympus BX61 microscope connected to Apple iMac computer ([www.apple.cp.uk](http://www.apple.cp.uk)). Analysis was performed using Image J v1.53e (https://imagej.nih.gov/ij) on a Dell personal computer (www.dell.co.uk).

For analysis of receptor expression on the circular muscle, three images of random areas were taken at x40 from each patient. αSMA-positive cells were examined for evidence of co-staining with V_1A_ and α_1A_ antibodies in the imaged region.

In preliminary experiments the ICC were differentiated from mast cells (both stained by c-Kit antibody^1^) by double staining for c-KIT and mast cell tryptase. The ICC were distinguished by the absence of staining by mast cell tryptase and as spindle-shaped cells with an oval nucleus and long projections; mast cells tended to have a rounder morphology to their nuclei and were stained by both c-KIT and mast cell tryptase (**Figure S7**). To select ICCs for analysis, numerous images were taken at x40 magnification in the circular muscle of each patient in which c-kit immunoreactive, spindle-shaped cells were apparent, selecting those images in which the ICC could be most clearly defined, as above. Three ICCs were analysed per patient (if more than 3 were observed, a random number generator selected cells to exclude from analysis). Analysed cells were classified as expressing the V_1A_ vasopressin and α_1_ adrenaline receptor if ≥50% of the cell body surrounding the nucleus was stained for the receptor.

Reference

1. Garrity MM, Gibbons SJ, Smyrk TC, et al. Diagnostic challenges of motility disorders: optimal detection of CD117+ interstitial cells of Cajal. Histopathol 2009;54:286-294.

**Antibodies used for immunohistochemistry**

| **Antibody** | **Concentration** | **Supplier** | **Catalogue code** |
| --- | --- | --- | --- |
| Goat c-Kit | 5x10^-3^ g/L | Bio-Techne, Abingdon, UK | AF1356 |
| Mouse mast cell tryptase | 1:800 | Agilent Dako, Stockport, UK | M705229-2 |
| Mouse Anti-alpha smooth muscle Actin | 6.8x10^-11^ g/L | Abcam, Cambridge, UK | AB7817 |
| Rabbit V_1A_ vasopressin receptor | 1:100 | Abcam, Cambridge, UK | AB140492 |
| Rabbit α_1_-adreneoceptor | 1:100 | Sigma-Aldrich, Gillingham, UK | A270 |
| Donkey Anti-Goat IgG H&L (Alexa Fluor® 488) | 1:500 | Abcam, Cambridge, UK | AB150129 |
| Donkey Anti-Rabbit IgG H&L (Alexa Fluor® 568) | 1:500 | Abcam, Cambridge, UK | AB175470 |
| Donkey Anti-Mouse IgG H&L (Alexa Fluor® 568) | 1:500 | Abcam, Cambridge, UK | AB175700 |

4′,6-diamidino-2-phenylindol (DAPI) was used at 5x10^-3^ g/L concentration. The auto-fluorescence quenching kit and coverslip mountant from Vector Laboratories, California, USA, and other reagents and materials from Sigma-Aldrich, Gillingham, UK.

**3. Method for analysing functional data**

Individual agonist concentration-response curves in the absence and presence of an antagonist or test ligand were fitted by non-linear regression to a four-parameter Hill equation (Equation 1) using GraphPad PRISM 7.0 for Windows (Graph-Pad Software, La Jolla, CA, USA), where E denotes response, log [A] the logarithm of the concentration of an agonist A, n_H_ the midpoint slope of the curve, log EC_50_ the logarithm of the midpoint location parameter along the concentration axis, and E_max_ and Basal the upper and lower asymptotes respectively.

Equation 1:

$$E=Basal+\frac{E_{max}-Basal}{1+{10}^{{(logEC}_{50}-log{[A])}^{n_{H}}}}$$

Rightward shifts of the agonist concentration-response curve by the presence of the antagonist were compared by a one-way ANOVA followed by a Dunnett’s *post hoc* test for multiple comparisons. The shifts were considered parallel if a sum-of-squares *F*-test on the Hill slope parameters of each family of the agonist concentration-response curves in the absence and presence of the antagonist indicated that the slope of each curve was not significantly different from unity. Additionally, if a sum-of squares *F*-test on the E_max_ parameter of the agonist concentration-response curves in the absence and presence of the competing ligand also showed no significant difference, each family of curves was re-fitted to Equation 1 with the E_max_ shared between each data set to a common value.

The concentration ratio for the rightward shift of the agonist concentration-response curve in the presence of a test ligand was determined as the ratio of concentrations corresponding to the EC_50_ response level of the curves (Equation 2).  When multiple concentrations of an antagonist were used, a Schild plot^1^ was constructed to obtain an estimate of the antagonist’s affinity from the negative logarithm of the equilibrium dissociation constant (*p*K_B_) of the antagonist-receptor complex. The *p*K_B_ represented the negative logarithm of the antagonist concentration that occupied 50% of the receptors at equilibrium.

If the line of best fit of the plot had a slope of unity (1.00), the antagonism was taken as being reversible competitive. However, if the slope was not equal to unity, but the 95% confidence intervals (CI) of the slope included unity, the regression was re-fitted to a unit slope to determine the *p*K_B_ of the antagonist. When a single value for the concentration ratio was available from experiments in which only a single concentration of antagonist was used, the antagonist potency (*p*A_2_) was calculated from the Gaddum–Schild equation (Equation 3):

Equation 2:

concentration ratio = $\frac{{Agonist EC}_{50} in presence of antagonist}{Agonist {EC}_{50} in absenece of antagonist}$

Equation 3

*p*A_2_ = log_10_ (concentration ratio -1) - log_10_ [antagonist]

References

1. Arunlakshana O, Schild HO. Some quantitative uses of drug antagonists. Br. J. Pharmacol. 1959;14:48–58.
2. Schild HO. pAx and competitive drug antagonism. Br J Pharmacol Chemother 1949;4:277-280.
