# Supplementary material for: Synergistic augmentation of rhythmic myogenic contractions of human stomach by arginine vasopressin and adrenaline: Implications for the induction of nausea
Source: Br J Pharmacol. 2022 Sep 22;179(24):5305–22. doi: 10.1111/bph.15943 (PMC9826163; doi:10.1111/bph.15943)
Supplement: Supplementary file 3 — Table S1: Anthropometric measurements and medical history of people donating stomach tissue. Table S2: (A) Potency (pEC50) and tissue maximal effect (Emax) of AVP for the increase in baseline muscle tension in the absence and presence of electrical field stimulation (EFS), and the amplitude and frequency of the spontaneous contractions of human proximal and distal stomach circular muscle when used fresh after resection or overnight (15–18 h) storage at 4°C in Krebs–Henseleit solution. (B) Amplitude of the EFS‐evoked contractions of fresh and overnight stored muscle strips before the application of AVP.Table S3: Potency (pEC50) and tissue maximal response (Emax) of AVP for the increase in baseline muscle tone, amplitude and frequency of the spontaneous contractions of human proximal and distal stomach circular muscle obtained from concentration‐response curves constructed using a cumulative and single dose per muscle strip dosing technique. Table S4. (A) Schild slopes and pKB values of SR49059 and L371257 for antagonism of respectively, AVP‐ and OT‐induced increase in muscle tone, amplitude and frequency of spontaneous contractions of human proximal and distal stomach circular muscle. (B) pA2 values of prazosin and yohimbine for the antagonism of the ADr‐induced changes in muscle tone and spontaneous contraction activity and inhibition of EFS‐evoked contractions of human proximal and distal stomach circular muscle. Table S5: Potency (pEC50) and tissue maximal response (Emax) of AVP for the increase in muscle tone, amplitude and frequency of the spontaneous contractions of human proximal and distal stomach circular muscle in the absence and presence of blockade of histamine H1 receptor and stabilisation of mast cells. Table S6. Amplitude of the electrical field stimulation (EFS)‐evoked contractions of human isolated proximal and distal stomach and when expressed as a fraction of the maximal contraction to carbachol (10−3 M), and the inhibitory effect of TTX (10−6 M) a [file BPH-179-5305-s001.docx]

**Table S1:** Anthropometric measurements and medical history of people donating stomach tissue.

| **No.** | **Sex** | **Age** | **Ethnicity** | **Weight (kg)** | **BMI (kg/m^2^)** | **Diabetic status** | **Class of medication consumed** | **Previous / Ongoing medical history** |
| --- | --- | --- | --- | --- | --- | --- | --- | --- |
| 1 | F | 59 | White | 146.8 | 59.6 | Type 2 | ACE inhibitor, Opioid analgesic, Antihyperglycemic, Statin | Hypertension, Diabetes, Hypercholesterolemia |
| 2 | M | 43 | White | 146.2 | 30+ | no | none | Malnutrition |
| 3 | M | 51 | Not stated | 241.6 | 75.0 | Type 2 | ACE inhibitor, Opioid analgesic, Ca^2+^ channel blocker, Antihyperglycemic, Diuretic | Hypertension, Diabetes, Sleep apnoea |
| 4 | F | 40 | No stated | 113.0 | 40.0 | no | none | Gall stones, Gastritis (*H.pylori*), Hypercholesterolemia |
| 5 | M | 55 | White | 128.4 | 40.5 | Type 2 | Statin, α_1A_ adrenoceptor antagonist | Hypercholesterolemia |
| 6 | F | 41 | Mixed | 129.4 | 47.0 | no | Opioid analgesic, Proton pump inhibitor, Diuretic, Thyroxine analog, Vitamin B_9_ | Chronic pain syndrome, Acid reflux, Hypothyroidism |
| 7 | M | 48 | White | 142.6 | 48.2 | Type 2 | Selective serotonin receptor inhibitor, Antihyperglycemic, Thyroid hormone analog | Diabetes, Hypothyroidism, Depression |
| 8 | F | 31 | White | 124.5 | 46.3 | no | Selective serotonin receptor inhibitor, Opioid and non-opioid analgesics, Vitamin D, Antimuscarinic, NSAID, Proton pump inhibitor, Multivitamins | Depression |
| 9 | M | 28 | Not stated | 160.8 | 53.7 | no | none | none |
| 10 | F | 57 | Black | 131.2 | 52.6 | no | Diuretic, Analgesic adjuvant, Ca^2+^ channel blocker, Opioid and non-opioid analgesics, Opiate antagonist | Chest pain, Palpitations, Hypertension |
| 11 | F | 46 | White | 98.4 | 42.6 | no | none | none |
| 12 | F | 25 | White | 94.2 | 40.2 | no | none | Smoker |
| 13 | F | 45 | Not stated | 151.8 | 55.8 | no | Proton pump inhibitor, Thyroid hormone analog, ACE inhibitor, multivitamins | Hypertension, Hypothyroidism |
| 14 | F | 24 | Black | 111.9 | 42.0 | no | Ca^2+^ channel blocker, Vitamin D, iron supplement, Vitamin B_9_, Proton pump inhibitor, Non-opioid analgesic, Gastro protectant, Multivitamins | Hypertension |
| 15 | F | 52 | Not stated | 96.4 | 39.6 | no | Analgesic adjuvant, Ca^2+^ channel inhibitor, Topical NSAID. NSAID, Antimuscarinic | Hypertension, Back pain |
| 16 | F | 60 | White | 81.2 | 31.3 | Type 2 | Thyroid hormone analog, Opioid analgesic, Statin, Xanthine oxidase inhibitor, Antihyperglycemic | Type 2 diabetes, Sleep apnoea, Hypothyroidism, Hypercholesterolemia |
| 17 | F | 39 | White | 104.0 | 42.2 | no | none | none |
| 18 | F | 35 | Black | 122.0 | 45.9 | no | none | none |
| 19 | F | 23 | Black | 148.2 | 56.5 | no | Inhaled corticosteroid, Opioid analgesic, Anticoagulant, Iron supplement, Sugar-based laxative, H_1_ histamine antagonist, Antiemetic, Proton pump inhibitor, β_2_ adrenoceptor agonist bronchodilator, Antihyperglycemic | Polycystic ovary syndrome, pelvic pain |
| 20 | F | 51 | White | 124.2 | 57.5 | no | none | Sleep apnoea, previous cholecystectomy |
| 21 | F | 60 | Not stated | 119.2 | 53.0 | Type 1 | ACE inhibitor, Selective serotonin/noradrenaline reuptake inhibitor, Ca^2+^ channel blocker, Statin, Inhaled corticosteroid | Hypertension, Hypercholesterolemia, Asthma, Smoker |
| 22 | F | 34 | Not stated | 132.4 | 48.6 | no | Selective serotonin reuptake inhibitor | Fatty liver, depression |
| 23 | M | 49 | Not stated | 124.8 | 39.3 | Type 2 | NSAID oral wash, Proton Pump Inhibitor, Ophthalmic β adrenoceptor blocker, Non-opioid analgesic, ACE inhibitor, Joint viscosupplementation agent, Fibrate, β_1_ adrenoceptor antagonist, Vitamin D, Anticoagulants, Diuretic, Prostanoid analog, Statin | Chronic renal impairment, Myocardial infarction, Factor V Leiden genotype, Type 2 diabetes with multiple complications, Fatty liver, Hip replacement |
| 24 | F | 48 | White | 145.0 | 55.3 | no | Opioid analgesic, Selective serotonin reuptake inhibitor, Ca^2+^channel inhibitor | Hypertension, Depression, Pain related to previous fractures (radial head, scaphoid bone, lateral humeral epicondyle) |
| 25 | M | 38 | Asian | 109.2 | 29.0 | no | NSAID, Statin | Vitamin D deficiency |
| 26 | F | 36 | Not stated | 218.4 | 69.0 | Type 1 | NSAID, Statin, Atypical antipsychotic, Antihyperglycemic, Antidepressant | Gall stones, Cholecystectomy, Renal calculus, Polycystic ovarian syndrome, Osteoarthritis, Diabetes, Asthma, Acid reflux, Depression |
| 27 | F | 58 | Asian | 98.8 | 44.5 | no | none | Depression, Sleep Apnoea, Acid reflux |
| 28 | F | 60 | White | 106.5 | 38.7 | no | none | Hysterectomy, Caesarean, Sleep apnoea |
| 29 | F | 28 | Not stated | 122.0 | 43.7 | no | none | none |
| 30 | M | 55 | Not stated | 140.0 | 47.3 | Type 1 | Anticoagulant, Adjuvant and opioid analgesics, Statin, Vitamin B_9_, | Sleep apnoea, Total hysterectomy, Benign essential Hypertension, Mild diabetes |
| 31 | F | 48 | White | 112.0 | 43.2 | no | Immunosuppressant, H_1_ antagonist, Adjuvant and opioid analgesics, Antibiotic. | Knee replacement, Osteoarthritis (knee and spine), Depression, Anxiety, Caesarean section, Low back pain, Hypothyroid, Asthma, Hypercholesterolemia, Ex-smoker |
| 32 | F | 54 | White | 98.0 | 35.1 | no | Selective Serotonin Reuptake Inhibitor, Proton pump inhibitor, Thyroxine analog, Antiemetic, Vitamins | Previous gastric band, Adhesiolysis |
| 33 | F | 36 | Not stated | 188.8 | 62.4 | no | Opioid and non-opioid analgesic | Back pain, Sleep apnoea |
| 34 | F | 56 | White | 123.4 | 45.9 | Type 2 | none | Vitamin D deficiency, Diabetes Type 2, Hypertension, Osteoarthritis, IBS |
| 35 | F | 46 | Black | 113.0 | 44.1 | Type 2 | Multivitamins | Sleep apnoea, Total hysterectomy, Benign essential Hypertension, Mild diabetes Type 2 |
| 36 | M | 45 | Not stated | 170.2 | 30+ | no | ACE inhibitor, Selective Serotonin Reuptake Inhibitor, Proton pump inhibitor, Thyroxine analog | Depression, Sleep apnoea |
| 37 | F | 52 | White | 82.9 | 34.5 | no | Tricyclic antidepressants, Statin, Corticosteroid, Fluoroquinolones (antibiotic), Opioids analgesic, Vitamin D, Laxative, H_1_ antagonist, Proton Pump Inhibitor, β₂ adrenergic receptor agonist | Depression, Anxiety, Suicidality, Constipation (uses laxative as necessary) |
| 38 | F | 52 | Not stated | 126.0 | 48.0 | no | Corticosteroid, β₂ adrenergic receptor agonist, Selective Serotonin Reuptake Inhibitor, Vitamin B | Osteoarthritis (Knee And Spine), Osteopenia, Previous breast cancer |
| 39 | M | 61 | White | 154.0 | 47.0 | no | Statin, Ang II receptor antagonist, Sulphonamide diuretic, Ca^2+^channel blocker | Bilateral insertional Achilles tendinopathy, |
| 40 | F | 55 | White | 104.1 | 36.9 | no | Complementary therapy, Proton pump inhibitor, Thiazide diuretic, Statin, Adjuvant and non-opioid analgesics | Sciatica, Backache, Hypertension, Acid reflux, Snoring, Apnoea |
| 41 | F | 66 | White | 102.0 | 43.6 | Type 2 | Anticoagulant, Statin, Selective Serotonin Reuptake Inhibitor, Corticosteroid, Estrogen, Analgesic adjuvant, Proton Pump Inhibitor, Laxative | Wedge fracture to spine, Sleep apnoea, Depression |
| 42 | M | 53 | White | 133.8 | 40.0 | Type 2 | Statin, Selective Serotonin Reuptake Inhibitor, Opioid analgesic, Analgesic adjuvant, Antihyperglycemic, Ca^2+^ channel blocker, NSAID, Incretin mimetic, Proton pump inhibitor | Diabetes Type 2, Hypertension, Hypercholesterolemia, Arthritis, Vitamin D Deficiency, Previous appendectomy, anal Fistula. Ex-smoker, Snoring |
| 43 | F | 33 | White | 119.4 | 45.7 | no | β₂ adrenoceptor agonist bronchodilator | Mild Asthma |
| 44 | F | 36 | Asian | 88.0 | 36.2 | Type 2 | Antihyperglycemic, Antihistamine, Thyroxine analog, β₂ adrenergic receptor agonist bronchodilator | Asthma |
| 45 | F | 36 | White | 112.4 | 44.5 | no | Non-opioid analgesic, Vitamin B_12_ | Osteoarthritis, Acid reflux, Previously (Molar Pregnancy, Gallstones, OSA, Polycystic Ovarian Syndrome), Bursitis |
| 46 | F | 67 | Not stated | 124.6 | 43.1 | no | ACE inhibitor, Thyroxine analog, Statin, Ca^2+^channel blockers, Analgesic adjuvant, | Chronic kidney disease, Mild hypercalcaemia, hypoparathyroidism |
| 47 | F | 56 | White | 105.0 | 40.0 | no | β₂ adrenergic receptor agonist bronchodilator, disease-modifying ant rheumatic drugs, Statin, Selective Serotonin Reuptake Inhibitor, anticholinergic bronchodilators | Depression, Arthritis, Back pain, |
| 48 | F | 31 | Not stated | 183.6 | 62.1 | no | none | Apnoea, Snoring |
| 49 | F | 49 | White | 141.6 | 54.6 | no | Opioid analgesic, β adrenoceptor antagonist, Selective serotonin reuptake inhibitor, tricyclic antidepressant, oral contraceptive | Osteoarthritis (spine and shoulders), Sleep apnoea, Depression, Anxiety, Sciatica |
| 50 | F | 55 | White | 116.2 | 42.7 | no | Anticoagulant, β adrenoceptor antagonist, anti-fungal | Hypertension |
| 51 | M | 63 | White | [clinical details unavailable] | | | | |
| 52 | F | 49 | White | [clinical details unavailable] | | | | |
| 53 | F | 35 | Black | [clinical details unavailable] | | | | |
|  |  |  |  |  |  |  |  |  |

**Table S2**: (A) Potency (*p*EC_50_) and tissue maximal effect (E_max_) of AVP for the increase in baseline muscle tension in the absence and presence of electrical field stimulation (EFS), and the amplitude and frequency of the spontaneous contractions of human proximal and distal stomach circular muscle when used fresh after resection or overnight (15-18 h) storage at 4^0^C in Krebs-Henseleit solution. (B) Amplitude of the EFS-evoked contractions of fresh and overnight stored muscle strips before the application of AVP

**A**

|  | **Proximal** | | | | **Distal** | | | |
| --- | --- | --- | --- | --- | --- | --- | --- | --- |
|  | **Fresh** | | **Overnight stored** | | **Fresh** | | **Overnight stored** | |
|  | ***p*EC_50_** | **E_max_ (% )** | ***p*EC_50_** | **E_max_ (% )** | ***p*EC_50_** | **E_max_ (% )** | ***p*EC_50_** | **E_max_ (% )** |
| **Tension** | 9.7 ± 0.1 | 7.3 ± 0.2 | 9.3 ± 0.1 | 6.5 ± 0.1 | 9.5 ± 0.1 | 10.1 ± 0.3 | 9.4 ± 0.1 | 9.2 ± 0.2 |
| **Amplitude** | 9.5 ± 0.2 | 263.4 ± 10.8 | 9.6 ± 0.1 | 216.6 ± 8.1 | 8.7 ± 0.1 | 63.8 ± 2.6 | 9.2 ± 0.2 | 55.1 ± 2.2 |
| **Frequency** | 9.3 ± 0.3 | 30.7 ± 5.7 | 8.8 ± 0.2 | 25.0 ± 0.0 | 8.8 ± 0.2 | 34.9 ± 1.5 | 9.2 ± 0.2 | 33.3 ± 0.00 |
| **Tone during EFS** | 9.2 ± 0.1 | 4.8 ± 0.1 | 8.6 ± 0.11 | 5.3 ± 0.1 | 8.6 ± 0.1 | 6.0 ± 0.1 | 8.6 ± 0.1 | 6.8 ± 0.2 |

**B**

|  | **Proximal** | | **Distal** | |
| --- | --- | --- | --- | --- |
|  | **Fresh** | **Overnight stored** | **Fresh** | **Overnight stored** |
|  | **Amplitude (mN)** | **Amplitude (mN)** | **Amplitude (mN)** | **Amplitude (mN)** |
| **EFS contraction** | 21.7 ± 1.5 | 21.0 ± 2.2 | 20.7 ± 1.2 | 19.7 ± 2.3 |

Data are mean±S.E.M. n=3.

**Table S3:** Potency (*p*EC_50_) and tissue maximal response (E_max_) of AVP for the increase in baseline muscle tone, amplitude and frequency of the spontaneous contractions of human proximal and distal stomach circular muscle obtained from concentration-response curves constructed using a cumulative and single dose per muscle strip dosing technique.

| \|  \| **Proximal** \| \| \| \| **Distal** \| \| \| \| \| --- \| --- \| --- \| --- \| --- \| --- \| --- \| --- \| --- \| \|  \| **Cumulative** \| \| **Single dosing** \| \| **Cumulative** \| \| **Single dosing** \| \| \| **Effect on** \| ***p*EC_50_** \| **E_max_ (% )** \| ***p*EC_50_** \| **E_max_ (% )** \| ***p*EC_50_** \| **(% )** \| ***p*EC_50_** \| **E_max_ (% )** \| \| **Tone** \| 9.5 ± 0.2 \| 7.1 ± 0.2 \| 9.8 ± 0.1 \| 7.3 ± 0.1 \| 9.5 ± 0.1 \| 9.5 ± 0.2 \| 9.7 ± 0.1 \| 10.1 ± 0.2 \| \| **Amplitude** \| 9.5 ± 0.1 \| 244.8 ± 3.9 \| 9.7 ± 0.1 \| 297.8 ± 7.7 \| 9.0 ± 0.1 \| 55.0 ± 2.0 \| 8.9 ± 0.2 \| 84.0 ± 3.9 \| \| **Frequency** \| 8.9 ± 0.1 \| 30.4 ± 2.6 \| 9.6 ± 0.1 \| 42.4 ± 3.0 \| 9.0 ± 0.1 \| 33.6 ± 1.0 \| 9.5 ± 0.1 \| 35.9 ± 0.9 \| |
| --- | --- | --- | --- | --- | --- | --- | --- | --- | --- | --- | --- | --- | --- | --- | --- | --- | --- | --- | --- | --- | --- | --- | --- | --- | --- | --- | --- | --- | --- | --- | --- | --- | --- | --- | --- | --- | --- | --- | --- | --- | --- | --- | --- | --- | --- | --- | --- | --- | --- | --- | --- | --- | --- | --- |

Data are mean±S.E.M. n=4-5.

**Table S4.** (A) Schild slopes and *p*K_B_ values of SR49059 and L371257 for antagonism of respectively, AVP- and OT-induced increase in muscle tone, amplitude and frequency of spontaneous contractions of human proximal and distal stomach circular muscle. (B) *p*A_2_ values of prazosin and yohimbine for the antagonism of the ADr-induced changes in muscle tone and spontaneous contraction activity and inhibition of EFS-evoked contractions of human proximal and distal stomach circular muscle.

**A**

|  |  | **SR49059 *vs* AVP** | | | **L371257 *vs* OT** | | |
| --- | --- | --- | --- | --- | --- | --- | --- |
| **Region** | **Effect on** | **Schild slope** | ***p*K_B_** | **True *p*K_B_**  **(slope = 1.0)** | **Schild slope** | ***p*K_B_** | **True *p*K_B_**  **(slope = 1.0)** |
| **Proximal** | **Tone** | 0.9 ± 0.1  (0.7 – 1.1) | 9.6 ± 0.2  (9.2 – 9.9) | 9.3 ± 0.2  (8.9 – 9.7) | 0.8 ± 0.1  (0.6 – 1.0) | 8.8 ± 0.1  (8.6 – 9.0) | 8.7 ± 0.1  (8.5 – 8.9) |
|  | **Amplitude** | 1.1 ± 0.1  (0.9 – 1.3) | 9.3 ± 0.5  (8.3 – 10.3) | 9.5 ± 0.1  (9.3 – 9.7) | 0.9 ± 0.1  (0.7 – 1.1) | 8.8 ± 0.1  (8.6 – 9.0) | 8.7 ± 0.1  (8.5 – 8.9) |
|  | **Frequency** | 0.8 ± 0.1  (0.6 – 1.0) | 9.9 ± 0.4  (9.1 – 10.7) | 9.3 ± 0.2  (8.9 – 9.7) | 0.9 ± 0.2  (0.8 – 1.9) | 8.6 ± 0.1  (8.4 – 8.8) | 8.7 ± 0.1  (8.5 – 8.98) |
| **Distal** | **Tone** | 1.0 ± 0.2  (0.6 – 1.4) | 9.5 ± 0.2  (9.1 – 9.9) | 9.4 ± 0.2  (9.0 – 9.8) | 0.7 ± 0.1*  (0.6 – 0.8) | 8.9 ± 0.1  (8.7 – 9.1) | 8.5 ± 0.1  (8.3 – 8.7) |
|  | **Amplitude** | 0.9 ± 0.1  (0.7 – 1.1) | 9.9 ± 0.3  (9.3 – 10.5) | 9.6 ± 0.1  (9.4 ± 9.8) | 0.9 ± 0.1  (0.8 – 1.0) | 8.4 ± 0.1  (8.2 – 8.6) | 8.8 ± 0.1  (8.6 – 9.0) |
|  | **Frequency** | 0.7 ± 0.1*  (0.5 – 0.9) | 9.7 ± 0.3  (9.1 – 10.3) | 9.5 ± 0.2  (9.1 – 9.9) | 0.9 ± 0.1  (0.8 – 1.0) | 8.7 ± 0.1  (8.5 – 8.9) | 8.6 ± 0.2  (8.4 – 9.1) |

Data are mean±S.E.M. n=5. * represents P<0.05. The 95% CI values in parenthesis.

**B**

|  |  | **Proximal** | **Distal** |
| --- | --- | --- | --- |
| **Antagonist** | **Effect** | ***p*A_2_** | ***p*A_2_** |
| **Prazosin** | **Tone (contraction)** | 9.5 ± 0.1 | 9.3 ± 0.2 |
|  | **Tone (relaxation)** | 7.5 ± 0.1 | 7.6 ± 0.2 |
|  | **Amplitude** | 9.3 ± 0.1 | 9.4 ± 0.1 |
|  | **Frequency** | 9.2 ± 0.4 | 9.6 ± 0.3 |
| **Yohimbine** | **EFS-amplitude** | 7.3 ± 0.2 | 7.5 ± 0.2 |

Data are mean±S.E.M. n=4

**Table S5:** Potency (*pEC*_50_) and tissue maximal response (E_max_) of AVP for the increase in muscle tone, amplitude and frequency of the spontaneous contractions of human proximal and distal stomach circular muscle in the absence and presence of blockade of histamine H_1_ receptor and stabilisation of mast cells.

|  | **Proximal** | | | | | | **Distal** | | | | | |
| --- | --- | --- | --- | --- | --- | --- | --- | --- | --- | --- | --- | --- |
|  | **Control** | | **+ Cromolyn** | | **+ Mepyramine** | | **Control** | | **+ Cromolyn** | | **+ Mepyramine** | |
| **Effect on** | ***p*EC_50_** | **Emax (%)** | ***p*EC_50_** | **Emax (%)** | ***p*EC_50_** | **Emax (%)** | ***p*EC_50_** | **Emax (%)** | ***p*EC_50_** | **Emax (%)** | ***p*EC_50_** | **Emax (%)** |
| **Tone** | 9.5 ± 0.1 | 7.3 ± 0.2 | 9.4 ± 0.1 | 7.4 ± 0.1 | 9.4 ± 0.1 | 7.2 ± 0.2 | 9.2 ± 0.1 | 8.5 ± 0.2 | 9.2 ± 0.1 | 7.8 ± 0.2 | 9.5 ± 0.1 | 8.1 ± 0.2 |
| **Amplitude** | 9.5 ± 0.1 | 256.7 ± 6.1 | 9.4 ± 0.1 | 243.4 ± 5.8 | 9.4 ± 0.2 | 250.6± 5.4 | 9.0 ± 0.1 | 68.5 ± 1.8 | 8.9 ± 0.1 | 69.8 ± 1.9 | 8.9 ± 0.1 | 68.2 ± 1.8 |
| **Frequency** | 9.1 ± 0.2 | 3.2 ± 0.0 | 9.2 ± 0.2 | 3.1 ± 0.1 | 9.2 ± 0.2 | 3.2 ± 0.0 | 8.9 ± 0.2 | 3.2 ± 0.0 | 8.8 ± 0.2 | 3.2 ± 0.0 | 8.9 ± 0.2 | 3.2 ± 0.1 |

Blockade of histamine H_1_ receptors or stabilisation of mast cells was achieved by incubation with mepyramine (10^-7^M) and cromolyn (10^-5^M), respectively, 30 min before examining AVP. Data are mean ±S.E.M. n=4

**Table S6.** Amplitude of the electrical field stimulation (EFS)-evoked contractions of human isolated proximal and distal stomach and when expressed as a fraction of the maximal contraction to carbachol (10^-3^M), and the inhibitory effect of TTX (10^-6^M) and atropine (10^-6^M).

| **Proximal** | | | | **Distal** | | | |
| --- | --- | --- | --- | --- | --- | --- | --- |
| **EFS-evoked contraction amplitude** | **Amplitude as % of carbachol-evoked contraction** | **% inhibition by TTX (10^-6^M)** | **% inhibition by atropine (10^-6^M)** | **Amplitude of EFS-evoked contraction** | **Amplitude as % of carbachol-evoked contraction** | **% inhibition of contraction by TTX (10^-6^M)** | **% inhibition of contraction by atropine (10^-6^M)** |
| 20.7 ± 0.8mN | 24.5 ± 2.1% | 97.9 ± 0.9% | 94.7 ± 1.5% | 19.8 ± 1.0mN | 27.2 ± 3.1% | 98.3 ± 1.5% | 99.3 ± 0.7% |

Atropine also caused a small decrease in muscle tone i.e relaxation of the both stomach regions during EFS (respectively, 1.2 ± 0.6mN and 0.9 ± 0.3mN). Data are mean±S.E.M. (n=3-16)

**Table S7:** Baseline and raised plasma concentrations of AVP and ADr in response to stimuli evoking nausea in human subjects

| **Subjects** | **Stimulus** | **Baseline [AVP]** | **Maximum [AVP]**  **Nausea** | **~Fold δ** | **Baseline**  **[ADr]** | **Maximum**  **[ADr] Nausea** | **~Fold Δ** | **Reference** |
| --- | --- | --- | --- | --- | --- | --- | --- | --- |
| **MOTION** | | | | | | | | |
| HV | Coriolis | 2.2±6.6pg/ml | 53.7±4.6pg/ml | x24 |  |  |  | 1 |
| HV | Vection |  |  |  | ~50pg/ml | >150pg/ml | x3 | 2 |
| HV | Vection | 2.55±0.36pg/ml | 38.4±28.9pg/ml  (one subject 183pg/ml) | x15 |  |  |  | 3 |
| HV | Vection | 1.73±1.01pg/ml | 123.1±29.5pg/ml  (individual values of >300pg/ml) | x71 | 62.8±8.9pg/ml | 140.4±32.5pg/ml | x2 | 4 |
| HV | Vection | 4.5±1.5pg/ml | 8.4±2.5pg/ml | x1.8 |  |  |  | 5 |
| HV | Vection | 2.1±0.5pg/ml | 5.3±0.4pg/ml | x2.5 |  |  |  | 6 |
| **PHARMACOLOGICAL CHALLENGES** | | | | | | | | |
| HV | Alcohol ingestion | ≤5pg/ml | 28-50pg/ml | x5-10 |  |  |  | 7 |
| HV | Apomorphine | 3.8±1.4pg/ml | 232.0±55.0pg/ml  (max individual value885pg/ml) | x61 |  |  |  | 7 |
| HV | Apomorphine | 0.4pg/ml (median) | 76pg/ml | x190 | 0.36nmol/l | 0.91nmol/l | x2.5 | 8 |
| HV | Apomorphine | 0.9±0.2pmol/l | 249±104pmol/l | x276 |  |  |  | 9 |
| HV | Apomorphine | <0.5pg/ml | ~7.5pg/ml | x49,  36,  13,  5 |  |  |  | 10 |
| P (hydrocephalus) | Apomorphine |  |  | x20-50 |  |  |  | 11 |
| HV | AVP infusion |  | 420±99pg/ml  (low dose; N=3)  687±89pg/ml  (high dose; N=4) |  |  |  |  | 12 |
| HV | AVP infusion | NS | 322.1±10.3pg/ml |  |  |  |  | 5 |
| HV | AVP infusion |  | 691.7pg/ml |  |  |  |  | 13 |
| HV | Cholecystokinin |  |  | x10 |  |  |  | 14 |
| HV | Ipecacuanha | Early basal 0.78±0.12pmol/l  Late basal 1.1±0.3pmol/ml | Early 21.7±9.4pmol/l  Late  5.68±2.55pmol/l  No change in 3 subjects reporting nausea but increase in others | x28 early  x6 late |  |  |  | 13 |
| HV | Nicotine | 4pg/ml | 26pg/ml | x6.5 |  |  |  | 15 |
| **ANTI-CANCER CHEMOTHERAPY** | | | | | | | | |
| P | Cancer chemotherapy | 5.53pg/ml | 33.83pg/ml  (post-emesis) | x6 |  |  |  | 16 |
| P | Cancer chemotherapy | 0.5-1.5pmol/l | 12.4±16.0pmol^-1^  (patients with V)  7.2 (0.4-52pmol^-1^)  (Patients with mod/severe N) | x4-129 |  |  |  | 17 |
| P | Cancer chemotherapy | 6.3±0.9ng/l | 15.1±3.3ng/l | x2.3 |  |  |  | 18 |
| **CLINICAL CONDITIONS** | | | | | | | | |
| P | Migraine | 0.5 (0.5-1.1) pg/ml | 3.5 (1.2-9.6) pg/ml | x7 |  |  |  | 19 |
| P | Tilt-induced syncope | ~100pg/ml | 676.5±122.8pg/ml | x6 | ~25pg/ml | 434±91.3pg/ml | x17 | 20 |

Changes in plasma AVP and ADr concentration in response to stimuli evoking nausea in healthy volunteers (HV) and patients (P). Note that the units of concentration are reported in the units used in the original publications but as these differ to facilitate comparison between publications the fold change (**Δ**) is also given as is the case for publications where the concentration values were not stated.

References

1. Eversmann T, Gottsmann M, Uhlich E, et al. Increased secretion of growth hormone, prolactin, antidiuretic hormone, and cortisol induced by the stress of motion sickness. Aviat Space Environ Med. 1978;49(1 Pt 1):53-7
2. Koch KL, Stern RM, Vasey MW, et al. Neuroendocrine and gastric myoelectrical responses to illusory self-motion in humans. Am J Physiol 1990a;258: E304-E310.
3. Koch KL, Summy-Long J, Bingaman S, et al. Vasopressin and oxytocin responses to illusory self-motion and nausea in man. J Clin Endocrinol Metab. 1990b;71(5):1269-75.
4. Xu LH, Koch KL, Summy-Long J, et al. Hypothalamic and gastric myoelectrical responses during vection-induced nausea in healthy Chinese subjects. Am J Physiol. 1993;265(4 Pt 1):E578-84
5. Kim MS, Chey WD, Owyang C, et al. Role of plasma vasopressin as a mediator of nausea and gastric slow wave dysrhythmias in motion sickness. Am J Physiol 1997;272: G853-G862.
6. Lien HC, Sun WM, Chen YH, et al. Effects of ginger on motion sickness and gastric slow-wave dysrhythmias induced by circular vection. Am J Physiol Gastrointest Liver Physiol. 2003;284(3):G481-9.
7. Rowe JW, Shelton RL, Helderman JH, et al. Influence of the emetic reflex on vasopressin release in man. Kidney Int. 1979;16(6):729-35.
8. Grant PJ, Hughes JR, Dean HG, et al. Vasopressin and catecholamine secretion during apomorphine-induced nausea mediate acute haemostatic function in man. Clin Sci (Lond).1986; 71: 621-624.
9. Nussey SS, Hawthorn J, Page SR, et al. Responses of plasma oxytocin and arginine vasopressin to nausea induced by apomorphine and ipecacuanha. Clin Endocrinol (Oxf). 1988;28(3):297-304.
10. Feldman M, Samson WK, O'Dorisio TM. Apomorphine-induced nausea in humans: release of vasopressin and pancreatic polypeptide. Gastroenterology. 1988;95(3):721-6.
11. Sørensen PS, Gjerris F, Hammer M. Cerebrospinal fluid and plasma vasopressin during short-time induced intracranial hypertone. Acta Neurochir (Wien). 1985;77(1-2):46-51.
12. Caras SD, Soykan I, Beverly V, et al. The effect of intravenous vasopressin on gastric myoelectrical activity in human subjects. Neurogastroenterol Motil. 1997;9(3):151-6. doi: 10.1046/j.1365-2982.1997
13. Page SR, Peterson DB, Crosby SR, et al. The responses of arginine vasopressin and adrenocrticotrophin to nausea induced by ipecacuanha. Clin Endocrinol (Oxf). 1990; 33:761-770.
14. Miaskiewicz SL, Stricker EM, Verbalis JG. Neurohypophyseal secretion in response to cholecystokinin but not meal-induced gastric distention in humans. J Clin Endocrinol Metab. 1989;68(4):837-43.
15. Goldsmith SR, Katz A, Crooks PA. Response of plasma arginine vasopressin to nicotine in normal man. Clin Pharmacol Ther.1988; 44(4): 478-481.
16. Fisher RD, Rentschler RE, Nelson JC, et al. Elevation of plasma antidiuretic hormones (ADH) associated with chemotherapy-induced emesis in man. Cancer Treat Rep. 1982;66(1):25-9.
17. Edwards CM, Carmichael J, Baylis PH, et al. Arginine vasopressin--a mediator of chemotherapy induced emesis? Br J Cancer. 1989;59(3):467-70.
18. Barreca T, Corsini G, Cataldi A, et al. Effect of the 5-HT_3_ receptor antagonist ondansetron on plasma AVP secretion: a study in cancer patients. Biomed Pharmacother. 1996;50(10):512-4
19. Hampton KK, Esack A, Peatfield RC, et al. Elevation of plasma vasopressin in spontaneous migraine. Cephalgia.1991;11:249-250.
20. Heyer GL, Boles LH, Harvey RA, et al. Gastric myoelectrical and neurohormonal changes associated with nausea during tilt-induced syncope. Neurogastroenterol Motil 2017; e13220.
